# The Compatibility of Astragali Radix and Angelicae Sinensis Radix Attenuates Atherosclerosis by Suppressing Vascular Endothelial PANoptosis via the p38 MAPK/p53 Pathway: A Multi-Omics and Experimental Study

**DOI:** 10.3390/ijms27188330

**Published:** 2026-09-19

**Authors:** Wanyu Li, Qingyin Long, Xinying Fu, Yanling Li, Lu Ma, Wei Tan, Zhongji Hu, Yanyan Zhang, Yan She, Wei Zhang, Changqing Deng

**Affiliations:** Key Laboratory of Hunan Provincial for Integrated Traditional Chinese and Western Medicine on Prevention and Treatment of Cardio-Cerebral Diseases, College of Integrated Chinese and Western Medicine, Hunan University of Chinese Medicine, Hunan 410208, China; liwanyu@stu.hnucm.edu.cn (W.L.);

**Keywords:** atherosclerosis, Astragali Radix, Angelicae Sinensis Radix, PANoptosis, vascular endothelial cells

## Abstract

Previous studies have reported that the combination of Astragali Radix and Angelicae Sinensis Radix (AR-ASR) can ameliorate atherosclerosis (AS) through anti-inflammatory effects; however, the exact mechanistic underpinnings still await further clarification. The present study investigates the anti-atherosclerotic action of AR-ASR from the perspective of PANoptosis (encompassing apoptosis, pyroptosis, and necroptosis). To ascertain the chief active ingredients of the AR plus ASR mixture, UPLC-MS/MS detection was utilized. To predict potential therapeutic targets, a multi-faceted bioinformatics strategy was applied, integrating transcriptomics, single-cell sequencing, network pharmacology, molecular docking, and molecular dynamics simulations. In vivo, male ApoE-deficient mice were allocated at random into the model cohort, AR-ASR low-, medium-, and high-dose groups (AR-ASR L, AR-ASR M, and AR-ASR H, respectively), atorvastatin group (ATV), and control group (age-matched wild-type C57BL/6J mice). Atherosclerosis was induced by 12 weeks of high-fat diet feeding, followed by 4 weeks of oral gavage treatment. Assessment parameters included aortic intimal hyperplasia thickness, serum lipid levels, vascular inflammatory factor expression, endothelial function, and the gene and protein expression levels of PANoptosis- and p38MAPK/p53 signaling pathway-related molecules. For in vitro experiments, POVPC-stimulated AVECs were used as an oxidative damage model and then treated with AR-ASR-containing serum. Cell proliferation activity, cell injury rate, and the expression levels of PANoptosis markers and p38MAPK/p53 signaling pathway-related molecules were measured. Furthermore, in the presence of a p38MAPK agonist, the protective effect of AR-ASR on AVECs and its underlying mechanism were investigated. Bioinformatics analysis suggested that AR-ASR modulates PANoptosis and interferes with AS via the p38MAPK/p53 signaling axis. In vivo, AR-ASR treatment reduced serum lipid levels, attenuated aortic intimal hyperplasia, decreased plasma inflammatory cytokine levels and vascular inflammatory factor expression, improved endothelial function, and suppressed both vascular PANoptosis and p38MAPK/p53 pathway activation. In vitro, POVPC induced oxidative damage and PANoptosis in AVECs. AR-ASR-containing serum promoted AVEC viability, reduced LDH release, inhibited the expression of PANoptosis-related markers, and downregulated p38MAPK/p53 signaling pathway protein expression. Furthermore, in the presence of a p38MAPK agonist, the inhibitory effects of AR-ASR on cellular PANoptosis and p38MAPK/p53 pathway activation were significantly attenuated. The AR-ASR compatibility suppresses p38MAPK/p53 pathway activation, thereby inhibiting vascular endothelial PANoptosis-driven inflammatory responses and ameliorating atherosclerotic vascular pathology.

## 1. Introduction

Cardiovascular afflictions continue to be a major global health strain, exhibiting notable prevalence, disability prevalence, and death prevalence [1]. Arterial-wall chronic inflammation, termed atherosclerosis, constitutes the main pathological basis of cardiovascular disorders [2]. AS pathogenesis is driven by an intricate network of processes, encompassing defective lipid regulation, oxidative insult, and inflammation of the arterial system [3]. Vascular endothelial injury serves as the initiating event and common pathological foundation of atherosclerosis; thus, counteracting endothelial injury and curbing inflammation within the vasculature are of substantial value in alleviating AS-related vascular abnormalities.

Vascular inflammatory reactions together with regulatory cell death (RCD) jointly shape the course of AS from its initiation through to its advancement [4,5]. Inflammation is a critical pathophysiological mechanism that persists throughout all stages of AS [6]. Meanwhile, RCD of vascular cells represents a major cause of vascular cell injury. RCD encompasses multiple forms, including apoptosis, pyroptosis, and necroptosis [7]. AS vasculopathy arises in part from deranged RCD activities of vascular cells, and blockade of these RCD routes is sufficient to produce substantial deceleration of AS development [8]. However, targeting a single RCD pathway does not substantially improve AS outcomes, which may be attributed to the crosstalk among different RCD modalities [9,10]. With in-depth investigation, the interplay among apoptosis, pyroptosis, and necroptosis was officially termed PANoptosis in 2019 [11]. Defined as an inflammatory RCD modality triggered by the PANoptosome, PANoptosis presents a triad of apoptotic, pyroptotic, and necroptotic signatures. Crucially, targeted inhibition limited to any one RCD pathway cannot effectively prevent PANoptosis from proceeding [11]. Vascular inflammation linked to AS is largely mediated through the action of PANoptosis [12]. Currently, most drugs targeting PANoptosis for AS treatment remain in the exploratory stage, and over-inhibition of a single target often carries risks such as immune dysfunction. Therefore, further exploration of strategies and agents that target PANoptosis is of great significance for the prevention and treatment of AS vasculopathy.

With its multi-agent and multi-pathway mode of action, Traditional Chinese Medicine (TCM) may offer regulatory intervention opportunities for PANoptosis. Qi insufficiency and blood stasis are recognized in TCM doctrine as the core pathogenic drivers underlying AS, with the clinical regimen of Qi-reinforcing and circulation-activating therapy proving to be a valid treatment option [13]. The compatibility of Astragali Radix and Angelicae Sinensis Radix (AR-ASR) exerts actions of tonifying Qi and activating blood circulation and is a classic herb pair in TCM for treating cardiovascular diseases. Its most famous formulation is Danggui Buxue Tang (DBT), with an AR-ASR ratio of 5:1, which has demonstrated favorable therapeutic efficacy against AS and other cardiovascular diseases [14]. Studies have shown that DBT exerts lipid-regulating, anti-inflammatory, and vascular endothelial cell-protective effects, thereby ameliorating AS-associated vascular pathology [15,16]. Our previous studies also demonstrated that AR-ASR at a 1:1 ratio suppresses vascular intimal hyperplasia and local inflammatory responses and inhibits RCD such as apoptosis in vascular wall cells [16]. However, whether AR-ASR compatibility ameliorates AS-associated vascular inflammation through inhibition of PANoptosis, and what the underlying mechanisms are, remain unclear. Given that AR-ASR compatibility inhibits both vascular inflammation and RCD of vascular wall cells, we hypothesize that AR-ASR may attenuate AS vasculopathy by regulating PANoptosis, a form of regulatory cell death in vascular cells, thereby suppressing inflammatory responses.

Therefore, in the present study, our research commenced with a multi-omics and bioinformatics strategy designed to explore the correlation between AS vasculopathy and PANoptosis, and to identify prospective targets of AR-ASR action. We then established an AS model in apolipoprotein E gene knockout (ApoE^−/−^) mice and an oxidative damage model in vascular endothelial cells, to investigate the anti-AS vascular protective effects of AR-ASR compatibility from the perspective of PANoptosis, and to elucidate its mechanism of action through the p38MAPK/p53 pathway, thereby providing experimental evidence for its clinical application.

## 2. Results

### 2.1. Quality Control of the Combination of AR-ASR

**Figure 1:**
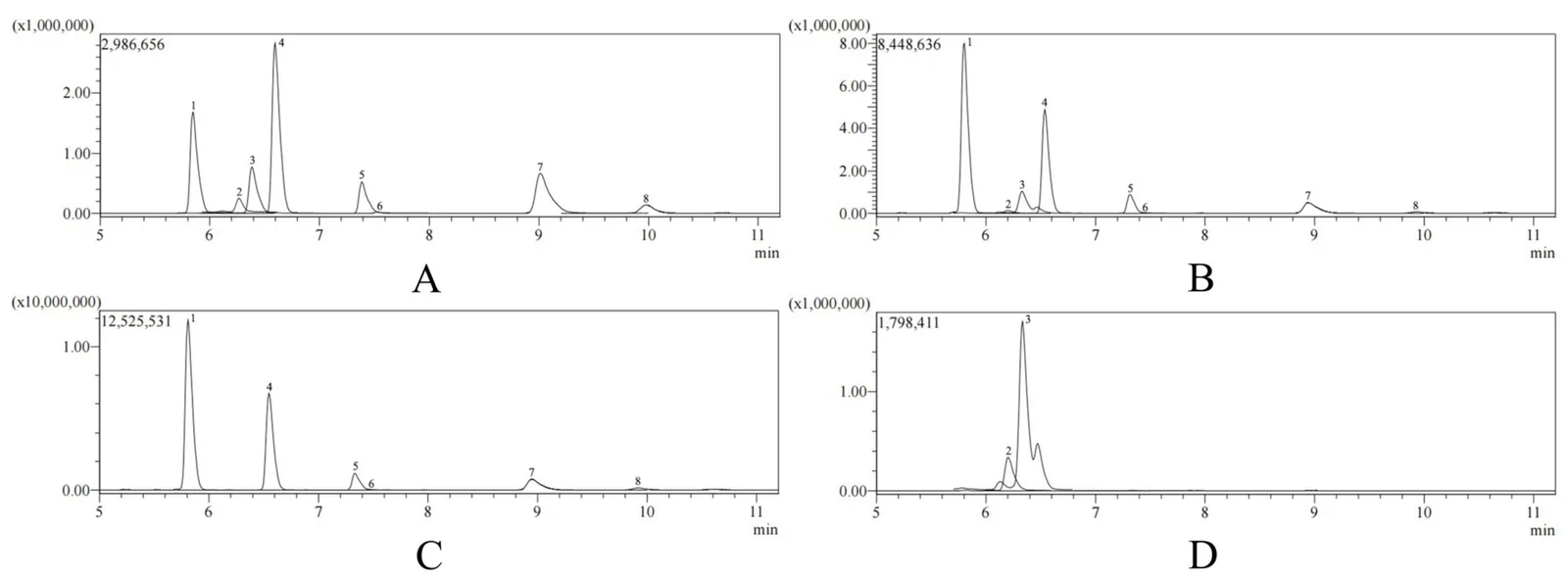
Identification of the main chemical components in the 1:1 combination of AR-ASR, as well as in the aqueous extracts of AR and ASR using UPLC-MS/MS. (**A**): mixed standards; (**B**): 1:1 combination of AR-ASR; (**C**): AR; (**D**): ASR. The standards are labeled as: 1: Calycosin-7-O-β-D-glucoside; 2: Ferulic acid; 3: Senkyunolide I; 4: Ononin; 5: Calycosin; 6: Astragaloside IV; 7: Formonnetin; 8: Astragaloside I.

The major chemical species in AR, ASR, and their 1:1 combination (all as aqueous extracts) were determined by means of UPLC-MS/MS. Comparison with reference standards confirmed that the 1:1 combination of AR-ASR contains calycosin-7-O-β-D-glucoside, ferulic acid, senkyunolide I, ononin, calycosin, astragaloside IV, formonnetin, and astragaloside I. AR contains calycosin-7-O-β-D-glucoside, ononin, calycosin, astragaloside IV, formonnetin, and astragaloside I. ASR contains ferulic acid and senkyunolide I (Figure 1).

### 2.2. AR-ASR Interferes with Atherosclerosis Progression by Specifically Regulating the p38MAPK/p53 Signaling Axis to Modulate PANoptosis

#### 2.2.1. Identification of DEGs in AS

**Figure 2:**
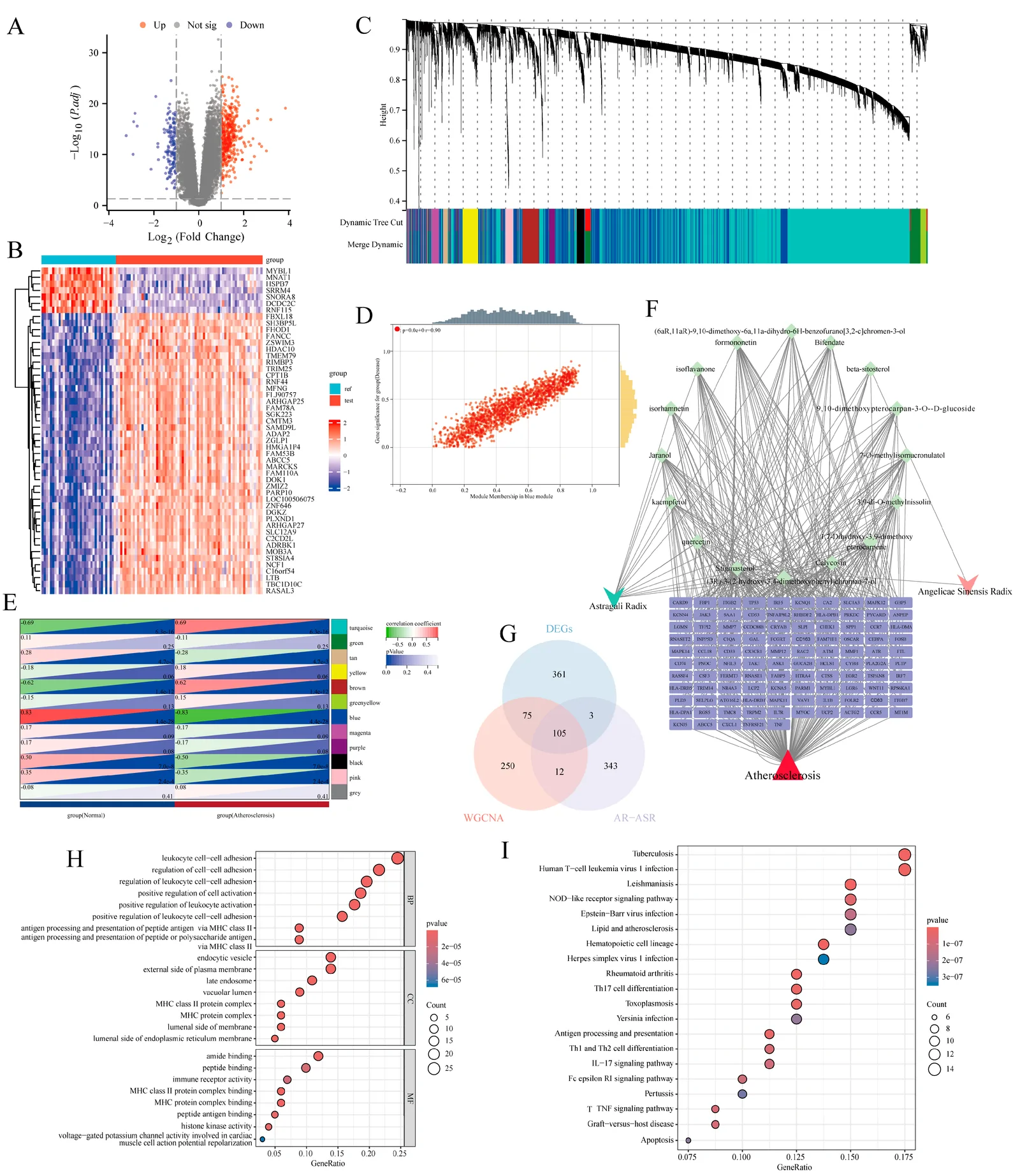
Screening and Functional Analysis of TTAA. (**A**) Volcano plot of DEGs in AS. (**B**) Heatmap of 544 DEGs in AS samples (experimental group) and control samples. (**C**) Sample dendrogram from WGCNA, used to identify and remove outlier samples. (**D**) Scatter visualization of the blue module, displaying the association between gene significance and module membership. (**E**) Heatmap of the correlation between gene modules and atherosclerosis clinical traits. (**F**) “Drug-Component-Target” interaction network of AR-ASR. (**G**) Venn depiction of the shared genes across AS DEGs, WGCNA core Hub genes, and probable AR-ASR drug targets. (**H**) Bubble visualization of GO enrichment results for the TTAA gene set. (**I**) KEGG enrichment analysis of TTAA is depicted as a bubble plot, wherein the *p*-value is encoded by bubble color and the enriched gene number is represented by bubble size.

DESeq2 was applied to the RNA-seq profiles of the GSE100927 dataset to identify genes showing differential expression between atherosclerotic and healthy arterial samples. With cutoffs set at adjusted *p* < 0.05 and |log_2_FC| > 1, 544 transcripts showed significant differential expression. In all, 544 DEGs were discovered (Figure 2A,B).

#### 2.2.2. WGCNA Identified Gene Modules Associated with AS

WGCNA revealed that the experimental group samples could be differentiated from the control group samples based on the overall transcriptional profile (Figure 2C). With β = 5 set as the soft-thresholding power, a scale-free network (R^2^ > 0.85) was generated, revealing 12 separate gene modules. Of these modules, the blue one correlated most strongly with key clinical traits of atherosclerosis, reaching r = 0.83 with *p* = 4.4 × 10^−28^ (Figure 2E). The module exhibited a notably strong positive MM-gene significance correlation (r = 0.90, *p* < 0.001), revealing that the genes contained in this network are critically involved in the development and worsening of AS (Figure 2D). Applying a stringent connectivity threshold (|MM| > 0.8), we selected 442 hub genes from this key network as primary candidates for subsequent exploration.

#### 2.2.3. Identification of Active Compounds in AR-ASR and Recognition of Their Core Therapeutic Targets

With OB ≥ 30% and DL ≥ 0.18 as the inclusion criteria, 16 potential active principles—including Stigmasterol and Mairin—were screened from the TCMSP database for the AR-ASR combination (Table 1). By integrating predictions from the PubChem (https://pubchem.ncbi.nlm.nih.gov/ (accessed on 8 September 2026)), SwissTargetPrediction (http://www.swisstargetprediction.ch/ (accessed on 8 September 2026)), PharmMapper (http://www.lilab-ecust.cn/pharmmapper/ (accessed on 8 September 2026)), and TargetNet (http://targetnet.scbdd.com/ (accessed on 8 September 2026)) databases, 463 potential protein targets associated with these active compounds were obtained. To further focus on targets most closely related to the pathological process of atherosclerosis, an intersection analysis was performed among these 463 drug targets, the previously identified DEGs, and the key Hub genes from WGCNA, resulting in the identification of 105 TTAA (Figure 2G). To elucidate the underlying regulatory mechanisms, a tripartite network depicting interactions among the drug, its bioactive constituents, and their molecular targets was constructed (Figure 2F), indicating that the AR-ASR combination may potentially treat AS through a multi-component, multi-target synergistic mechanism.

#### 2.2.4. GO and KEGG Functional Enrichment Analysis

The biological functions and prospective pathway associations of TTAA were further explored through GO/KEGG enrichment analysis of this gene set. GO analysis identified that TTAA was prominently enriched in several core immune-related biological processes, such as leukocyte cell–cell adhesion and positive regulation of cell activation. CC and MF analyses demonstrated that these target proteins resided mainly at the plasma membrane’s external side and the MHC class II complex, mediating key processes such as immune receptor activity (Figure 2H).

KEGG functional enrichment results provided additional evidence for the involvement of TTAA within particular signaling cascades. The results showed that the “Lipid and Atherosclerosis” pathway, directly related to disease pathology, was significantly enriched. Additionally, TTAA was significantly enriched in multiple core immune-inflammatory regulatory pathways, including the “NOD-like receptor signaling pathway”, the “TNF signaling pathway”, and the “Apoptosis” pathway. The results revealed that TTAA may participate in a complete regulatory cascade in the pathogenesis of atherosclerosis, ranging from inflammasome activation (closely associated with pyroptosis) and adaptive immune responses to PANoptosis (apoptosis, pyroptosis, and necroptosis) (Figure 2I). These results imply that AR-ASR’s therapeutic potential may stem from its combined modulation of immune-inflammatory reactions and PANoptosis.

#### 2.2.5. Core Role of TTAA in PANoptosis Pathways

**Figure 3:**
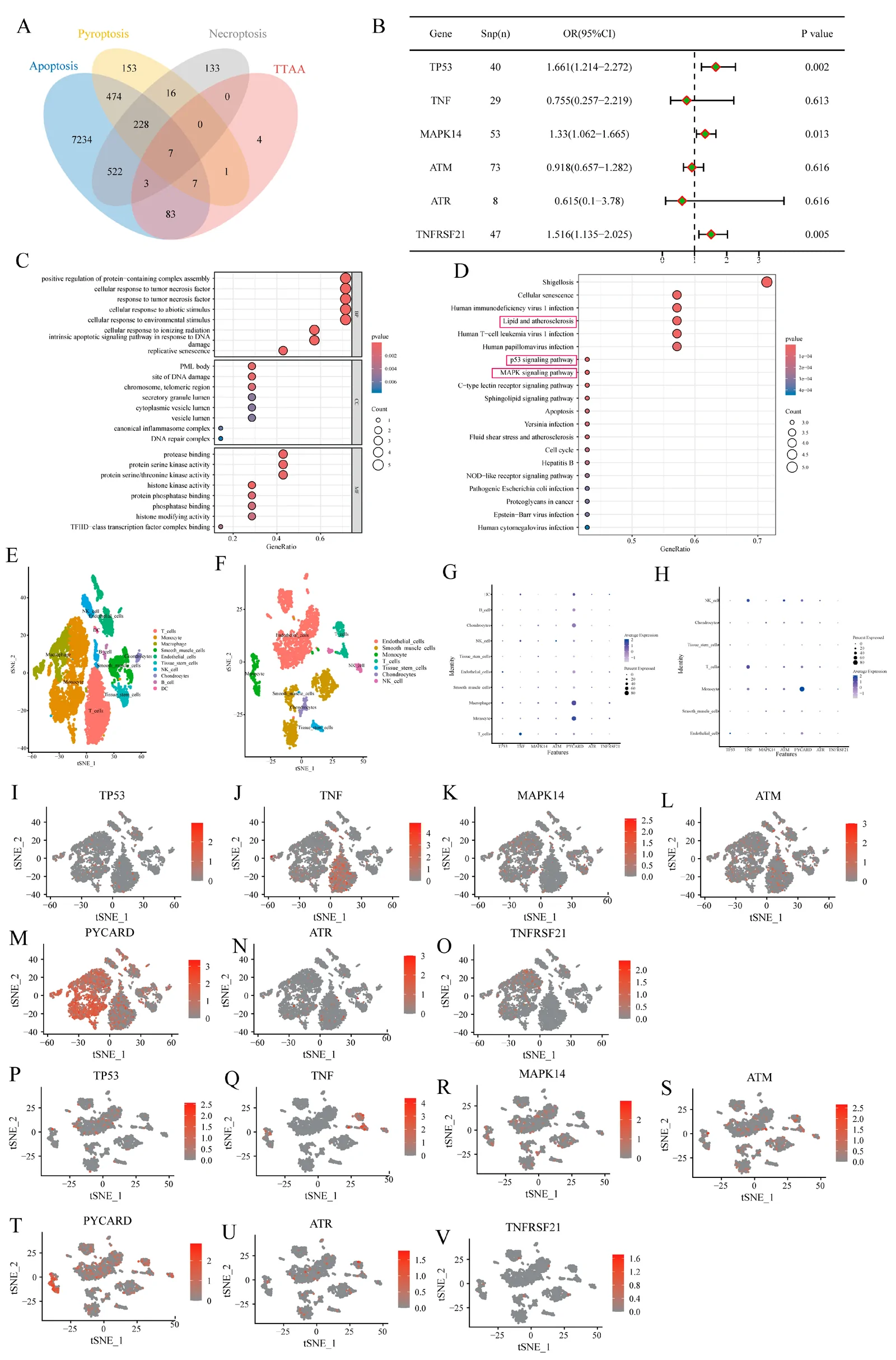
Identification, Functional Characterization, and Single-Cell Localization of ARCH Genes. (**A**) Overlap analysis via Venn diagram of the 105 TTAA genes (core AR-ASR targets for AS therapy) with apoptosis-, pyroptosis-, and necroptosis-related gene panels resulted in the final selection of 7 ARCH genes. (**B**) Presented is a forest plot from a two-sample MR study, showing the causal effect of ARCH gene expression on AS susceptibility. Squares mark the odds ratios, and horizontal lines denote the 95% confidence intervals. (**C**) GO functional enrichment of the ARCH genes, covering BP, CC, and MF ontologies. (**D**) KEGG pathway enrichment analysis of the ARCH gene set is presented. (**E**,**F**) t-SNE dimensionality reduction plots, showing the annotated cell clusters in (**E**) atherosclerotic core plaques (AC) and (**F**) adjacent paired tissues (PA). (**G**,**H**) Bubble visualization of ARCH gene expression across cell types in (**G**) AC and (**H**) PA tissues. (**I**–**V**) Feature plots showing the specific expression and spatial distribution of each individual ARCH gene in (**I**–**O**) AC tissue and (**P**–**V**) PA tissue. The genes shown in sequence are: (**I**,**P**) TP53, (**J**,**Q**) TNF, (**K**,**R**) MAPK14, (**L**,**S**) ATM, (**M**,**T**) PYCARD, (**N**,**U**) ATR, and (**O**,**V**) TNFRSF21. The red intensity in the plots is positively correlated with gene expression levels.

A seven-gene ARCH (TP53, TNF, MAPK14, ATM, PYCARD, ATR, and TNFRSF21) was identified through an intersecting study between 105 TTAA and core genes of the apoptosis, pyroptosis, and necroptosis pathways in order to ascertain the relevance of TTAA in controlling cell death. Functional enrichment analysis of ARCH was then conducted to reveal its more precise biological mechanisms (Figure 3A).

This gene collection is markedly enriched in cellular stress response processes at the BP level, such as “response to tumor necrosis factor” and “intrinsic apoptotic signaling pathway,” according to the GO analysis results. The CC analysis indicated that these target proteins are localized in key signaling complexes, including the “canonical inflammasome complex” and “DNA repair complex”. MF analysis primarily involved enzyme activities regulating signal transduction, such as “protein serine/threonine kinase activity” (Figure 3C).

KEGG enrichment analysis identified significant pathway enrichment in stress response, p53 signaling, and MAPK signaling, which highly coincides with concurrent enrichment in cellular senescence, apoptosis, and cell cycle. Notable enrichment was also found in NOD-like receptor signaling and lipid and atherosclerosis. These data imply that the seven core targets form a crucial molecular node linking external stress signals, p53/MAPK signaling transduction, PANoptosis, and the pathophysiological processes of atherosclerosis (Figure 3D).

#### 2.2.6. Causal Effect Assessment of ARCH on AS

To evaluate the potential causal contribution of ARCH genes to atherosclerotic pathogenesis, a two-sample MR analysis was performed on this gene collection. Due to the lack of a valid instrumental variable for the PYCARD gene in the eQTLGen database, causal inference was conducted on the remaining six genes (TP53, TNF, MAPK14, ATM, ATR, and TNFRSF21).

MR results indicated that genetically predicted expression of three ARCH genes was causally linked to atherosclerotic disease risk. Higher genetically predicted expression of TP53 (OR = 1.661, 95% CI = 1.214–2.272, *p* = 0.002), MAPK14 (OR = 1.330, 95% CI = 1.062–1.665, *p* = 0.013), and TNFRSF21 (OR = 1.516, 95% CI = 1.135–2.025, *p* = 0.005) significantly increased atherosclerosis risk. Conversely, the expression levels of TNF, ATM, and ATR showed no statistically significant causal relationship with atherosclerosis susceptibility (*p* > 0.05) (Figure 3B).

The reliability of the causal estimates was corroborated by sensitivity checks. No appreciable heterogeneity emerged from Cochran’s Q test for TP53, MAPK14, and TNFRSF21, and the absence of horizontal pleiotropy was confirmed by both MR-Egger intercept and MR-PRESSO (*p* > 0.05), reinforcing the overall validity of the findings.

#### 2.2.7. Cell-Specific Expression Atlas of ARCH Genes in the AS Plaque Microenvironment

Single-cell transcriptome data from the patients’ core atherosclerotic plaque (AC) and adjacent paired tissue (PA) were analyzed to determine the single-cell expression patterns of ARCH genes. Ten different cell clusters, including T cells, monocytes, macrophages, smooth muscle cells, endothelial cells, and trace amounts of B cells and dendritic cells, were found in AC tissue, according to cell type annotation data (Figure 3E), whereas PA tissue mainly consists of seven cell types, lacking macrophages, B cells, and dendritic cells (Figure 3F). The expression localization of the seven ARCH genes was systematically evaluated based on this cell atlas. In AC tissue, the expression of TP53 and MAPK14 was predominantly localized to monocytes, macrophages, and endothelial cells. TNF expression was mainly found in T cells, with some expression in monocytes. ATM expression was observed in T cells, macrophages, and monocytes. PYCARD transcript levels were notably elevated in T cells, monocytes, and macrophages, and a high percentage of cells residing in these clusters expressed this gene. TNFRSF21 expression was mainly confined to monocytes and macrophages. In contrast, ATR expression was not significantly observed in any cell cluster (Figure 3I–O). In contrast, PA tissue exhibited notably different expression patterns: TP53 expression was predominantly restricted to endothelial cells. TNF expression was mainly observed in T cells, monocytes, and NK cells. ATM expression was primarily distributed in monocytes and NK cells. PYCARD expression was widely distributed across endothelial cells, monocytes, T cells, chondrocytes, and NK cells. MAPK14, ATR, and TNFRSF21 showed no significant expression across any of the cell types in PA tissue (Figure 3P–V). Altogether, this analysis established substantial cell-population-specific differences in ARCH expression between the lesion core and the neighboring tissue. Monocytes, macrophages, T cells, and endothelial cells were identified as pivotal orchestrators of major PANoptosis-related gene expression changes, especially within the central portion of the atherosclerotic plaque. The expression level bubble plots are shown in Figure 3G,H.

#### 2.2.8. Molecular Docking of Major Active Components with Targets

**Figure 4:**
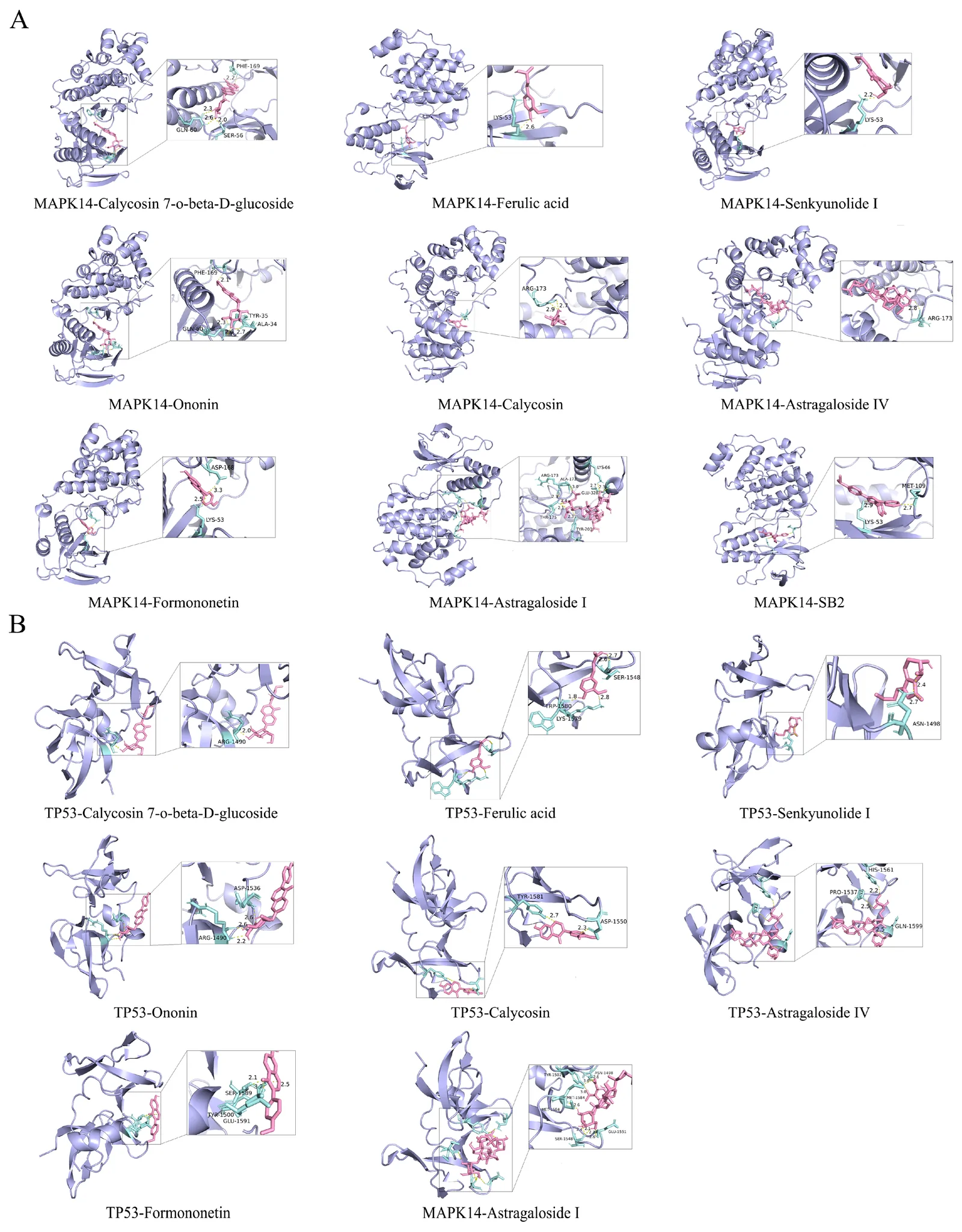
Visualization of molecular docking between core targets and active components. (**A**) Depiction of TP53 docking with each bioactive molecule; (**B**) visualization of MAPK14 docking with each active component and reference molecule.

From the above findings, we docked the screened ARCH against the main active compounds of AR-ASR (Table 2). In accordance with the literature [17], binding energies of <−4.25, <−5.0, and <−7.0 kcal·mol^−1^ denote binding activity, good binding activity, and strong binding activity, respectively. The binding activity results demonstrated that the predominant chemical agents in AR-ASR—including calycosin-7-O-β-D-glucoside, ferulic acid, senkyunolide I, ononin, calycosin, astragaloside IV, formononetin, and astragaloside I—displayed favorable binding to the ARCH candidates. Combined with the analyses in sections “Core Role of TTAA in PANoptosis Pathways” and “Causal Effect Assessment of ARCH on AS”, we identified TP53 and MAPK14 as the core targets closely associated with PANoptosis. The docking conformations of the major active ingredients and reference standards were rendered visually in PyMOL 2.5.0 (Figure 4).

#### 2.2.9. MD Simulation Analysis of Major Active Components Binding to Targets

**Figure 5:**
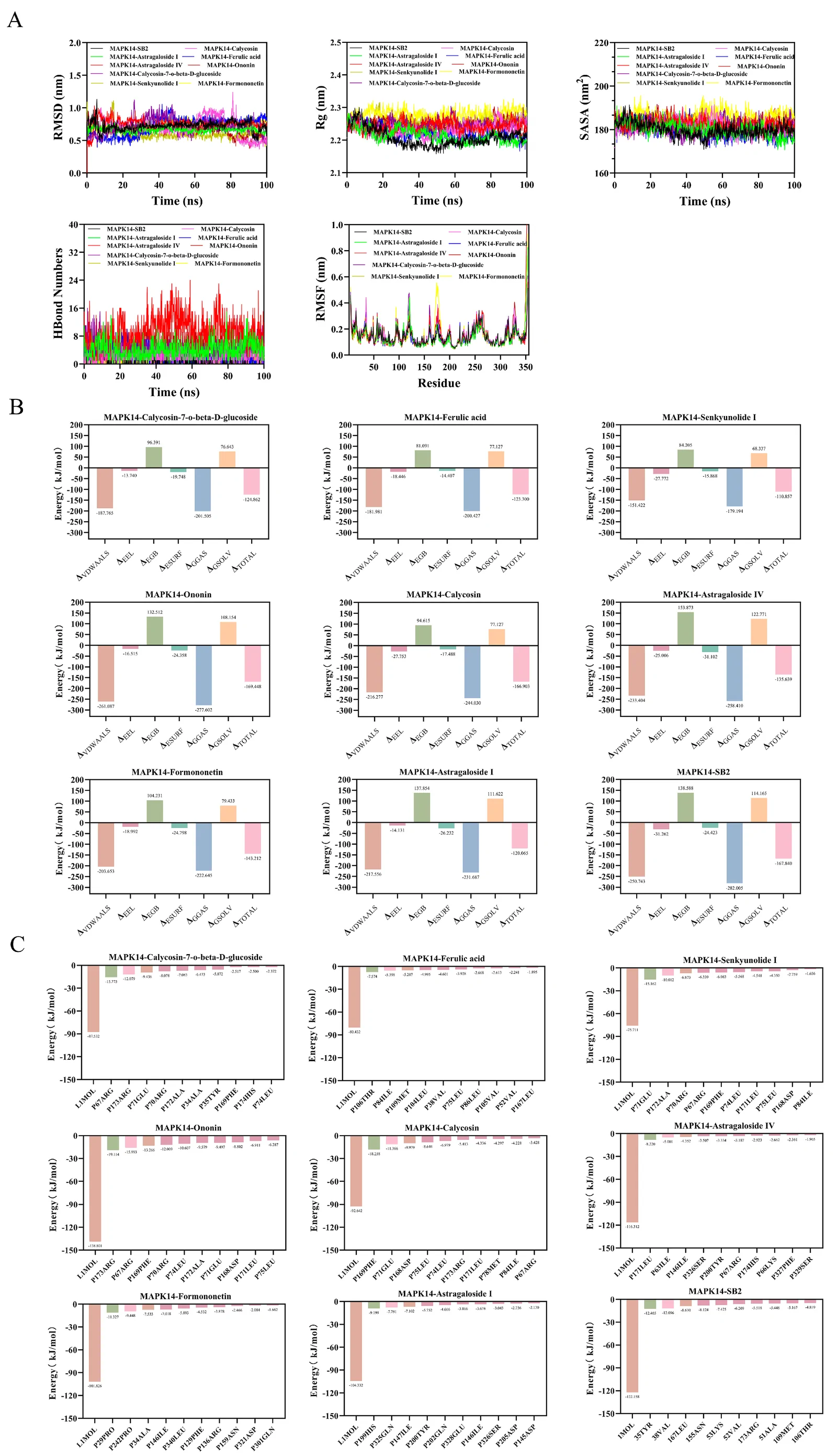
Molecular dynamics simulation of MAPK14 with each active component. (**A**) MD simulation trajectory visualization; (**B**) illustration of the total binding free energy with its constituent energy terms; (**C**) breakdown analysis of the system’s energy contributions.

Through molecular docking analysis, we identified MAPK14 and TP53 as the core targets with strong binding activity to the major active components. Since MAPK14 mediates the activation of p53, MAPK14 was selected as the primary core target, and the major active components of AR-ASR, including calycosin-7-O-β-D-glucoside, ferulic acid, Senkyunolide I, ononin, calycosin, astragaloside IV, formonnetin, and astragaloside I, were selected as the research subjects. To make docking results more interpretable, we used SB2 (a known ligand for MAPK14, −8.8 kcal/mol) as a reference (Figure 4A) and performed MD simulation to evaluate protein-ligand complex stability and interaction profiles (Figure 5).

RMSD values of all complex systems were largely confined to 0.5–1.0 nm, reflecting stable states and a consistently stable trend for most systems during the simulation. Among them, the MAPK14-Senkyunolide I (yellow curve) and MAPK14-SB2 (black curve) complexes exhibited the most stable behavior, with RMSD values consistently maintained at a low level (approximately 0.6 nm), suggesting optimal binding stability. In contrast, the MAPK14-Calycosin complex (pink curve) showed obvious fluctuations in the later stage of the simulation (around 80 ns), with RMSD peaks exceeding 1.0 nm, indicating that substantial adjustments in its binding conformation may have occurred. According to overall RMSD results, most ligands maintained dynamic equilibrium with MAPK14 over 100 ns, indicating stable binding comparable to or more stable than the reference molecule. Radius of gyration (Rg) analysis showed that the Rg values of all complexes were essentially maintained within the range of 2.15–2.30 nm with small fluctuations, indicating that the overall conformation of the MAPK14 protein remained well compacted after binding to different ligands. The Rg value of the MAPK14-SB2 complex (black) showed a slight decreasing trend, indicating a more compact structural feature, while other systems remained relatively constant. Solvent accessible surface area (SASA) analysis showed that the overall SASA values ranged from 170 to 190 nm^2^, with no significant drastic fluctuations throughout the 100 ns simulation, indicating that the complexes maintained stable surface areas in the aqueous environment without obvious protein unfolding or abnormal aggregation. Each system exhibited notably distinct hydrogen bond counts, as revealed by subsequent hydrogen bond analysis. Among them, the MAPK14-Astragaloside IV complex (red curve) exhibited the most prominent performance, with the number of hydrogen bonds significantly higher than that of the other systems, fluctuating between 8 and 24, and lasting for a long duration, suggesting the presence of a strong hydrogen bond network interaction between this ligand and the MAPK14 protein, which may be an important source of its high affinity. The MAPK14-Calycosin-7-O-β-D-glucoside complex (purple) and MAPK14-Astragaloside I complex (green) followed, maintaining approximately 4–10 hydrogen bonds. In contrast, small molecule ligands such as ferulic acid and formononetin formed relatively fewer hydrogen bonds (0–5), suggesting that their binding may rely more on hydrophobic interactions or other non-covalent interactions. In summary, each ligand engaged in stable, abundant hydrogen bonding with the target protein. RMSF analysis further indicated small residue fluctuations (mostly 0.1–0.3 nm) and low core region flexibility, implying high stability of key binding sites during simulation (Figure 5A).

Figure 5B presents the MM/PBSA-calculated total binding free energy and the contribution of individual energy components. The results demonstrated that the major active components of AR-ASR, including calycosin-7-O-β-D-glucoside, ferulic acid, Senkyunolide I, ononin, calycosin, astragaloside IV, formonnetin, and astragaloside I, all exhibited strong affinity for the MAPK14 target (calycosin-7-O-β-D-glucosidee: −124.862 kJ/mol; ferulic acid: −123.300 kJ/mol; senkyunolide I: −110.857 kJ/mol; ononin: −169.448 kJ/mol; calycosin: −166.903 kJ/mol; astragaloside IV: −135.639 kJ/mol; formononetin: −143.212 kJ/mol; astragaloside I: −120.065 kJ/mol). These observations imply that the active components have stable target binding and promising bioactivity.

To better characterize the interaction modes between MAPK14 and the major active components, we applied energy decomposition analysis to the target protein and its assembled complexes (Figure 5C). The results showed that ononin (−138.801 kJ/mol) exhibited the most significant binding to MAPK14, with binding energy superior to the reference molecule SB2. The other active components likewise showed potent binding, with Arg173, Leu171, and His199 as key energy contributors, indicating potential advantages for MAPK14 activity modulation.

In summary, all major AR-ASR active components exhibited strong stability and MAPK14 affinity. MD and MM/PBSA results confirmed stable complex formation, conformational adaptability, and energetic matching. These findings suggest potential PANoptosis-targeted intervention and support further mechanistic studies of AR-ASR.

### 2.3. The AR-ASR Combination Inhibited Vascular Intimal Hyperplasia, Reduced Serum Lipid Levels, and Improved Vascular Function and Inflammatory Responses in the AS Model

**Figure 6:**
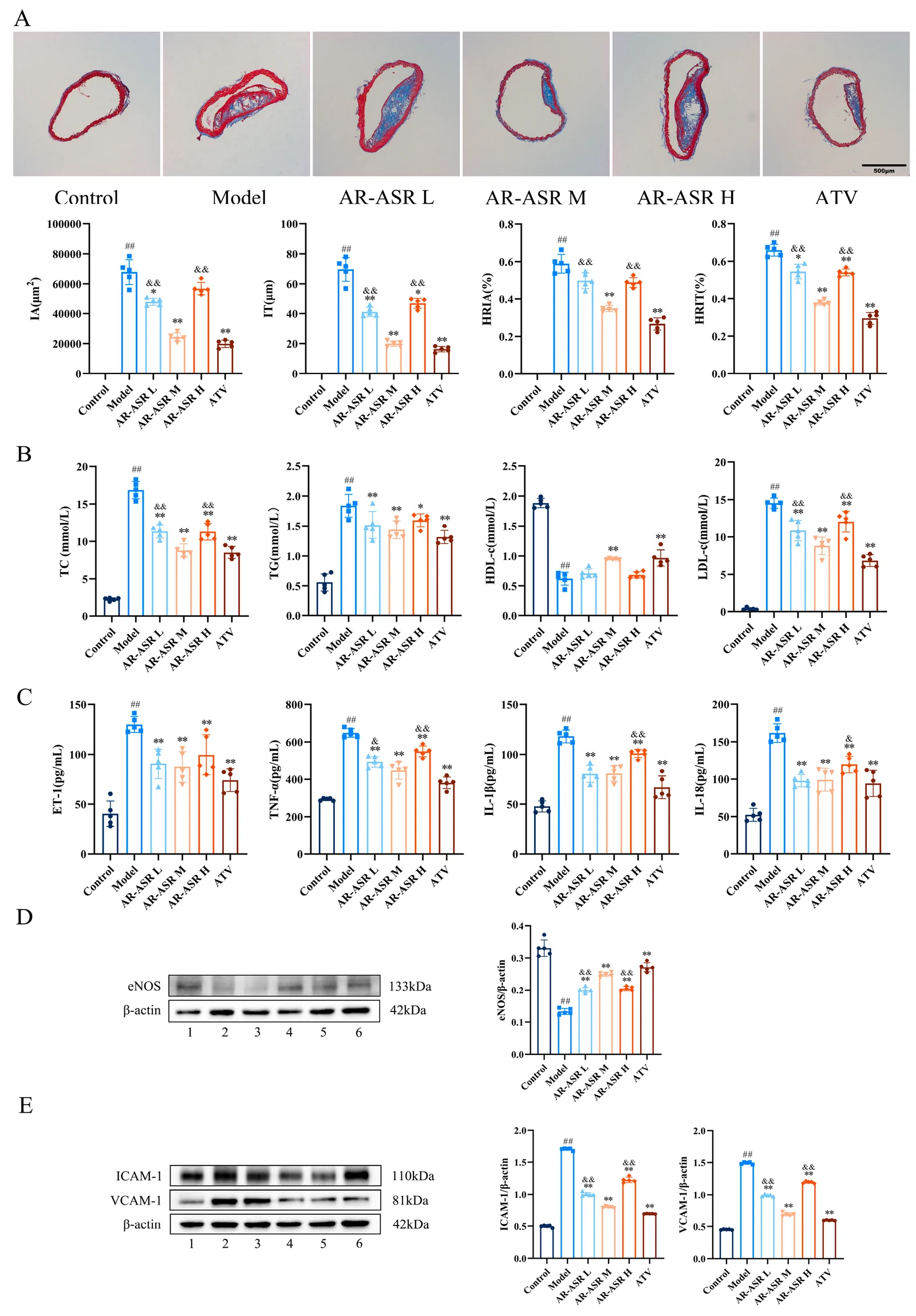
Effects of AR-ASR Combination on Vascular Intimal Hyperplasia, Blood Lipids, Endothelial Function, and Inflammatory Responses in AS Mice ($\bar{x}$ ± *s*, *n* = 5). (**A**) Evaluation of vascular intimal growth responses; (**B**) comparison of blood lipids; (**C**) comparison of plasma ET-1, TNF-α, IL-1β, and IL-18 levels; (**D**) comparison of eNOS expression in the aorta; (**E**) intergroup comparison of aortic ICAM-1 and VCAM-1 protein levels. Control: Control group; Model: Model group; AR-ASR L: Low-dose AR-ASR combination group; AR-ASR M: Medium-dose AR-ASR combination group; AR-ASR H: High-dose AR-ASR combination group; ATV: Atorvastatin group. 1: Control; 2: Model; 3: AR-ASR L; 4: AR-ASR M; 5: AR-ASR H; 6: ATV. Compared to the control group: ^##^ *p* < 0.01; compared to the model group: ** *p* < 0.01, * *p* < 0.05; compared to the medium-dose AR-ASR group: ^&&^ *p* < 0.01, ^&^ *p* < 0.05.

The initial phase of the study evaluated the impact of the AR-ASR combination on key parameters in AS models, including vascular intimal hyperplasia, blood lipid profiles, vascular function, and systemic inflammation. Aortic morphometry demonstrated that the model group exhibited significant increases in intimal area (IA), intimal thickness (IT), and the ratios of intimal to medial area (HRIA) and thickness (HRIT) (*p* < 0.01). The hyperplastic parameters were substantially lowered by AR-ASR administration when set against the model controls (*p* < 0.05). The medium-dose group also exhibited more substantial lowering of IA, IT, HRIA, and HRIT than did either the low- or high-dose cohorts (*p* < 0.01), as illustrated in Figure 6A. Lipid analysis showed that the model group had significantly higher plasma TC, TG, and LDL-c than the control group (*p* < 0.01), while HDL-c was substantially diminished, while HDL-c was significantly reduced (*p* < 0.01). Both AR-ASR and ATV treatment effectively diminished TC, TG, and LDL-c in comparison with the model group (*p* < 0.05). HDL-c was significantly raised in both the medium-dose AR-ASR and ATV groups (*p* < 0.01). The medium-dose AR-ASR group exhibited significantly decreased TC and LDL-c in contrast to both the lower and higher dosage groups (*p* < 0.01), as presented in Figure 6B. As a peptide chiefly derived from vascular endothelial cells, ET-1 functions as a critical regulator of vasoconstriction and cardiovascular balance. TNF-α, IL-1β, and IL-18 serve as markers of systemic inflammatory status. This investigation revealed that the model group had significantly greater ET-1 concentrations than the control group (*p* < 0.01). Administration of AR-ASR across all tested doses, as well as ATV treatment, effectively reduced circulating ET-1 levels relative to the model group (*p* < 0.01). Similarly, the model cohort exhibited considerable elevations in plasma TNF-α, IL-1β, and IL-18 relative to controls (*p* < 0.01). Either AR-ASR or ATV treatment effectively reduced these heightened cytokine levels (*p* < 0.01). It is noteworthy that administration of AR-ASR at the medium dosage produced a more pronounced decrease in TNF-α levels than either the lower or higher dosage groups (*p* < 0.05, Figure 6C). In aggregate, these findings demonstrate that AR-ASR co-administration reduces arterial intimal thickening, normalizes blood lipids, and improves vascular function in the atherosclerosis model.

eNOS, mainly expressed by vascular endothelial cells, indicates endothelial function. Meanwhile, ICAM-1 and VCAM-1, predominantly localized to the vascular endothelial surface, mediate leukocyte adhesion; their upregulation signals vascular wall inflammation. eNOS protein levels in the model cohort were found to be markedly lower than those in the control group, according to our findings (*p* < 0.01). eNOS expression was significantly higher in the AR-ASR low-, medium-, and high-dose groups and the ATV group than in the model group (*p* < 0.01). Notably, the medium-dose AR-ASR group showed significantly higher eNOS expression than the low- and high-dose groups (*p* < 0.01), as shown in Figure 6D. Relative to the control animals, the model group exhibited markedly elevated protein levels of both ICAM-1 and VCAM-1 (*p* < 0.01). ICAM-1 and VCAM-1 expression was significantly lower in the AR-ASR low-, medium-, and high-dose groups and the ATV group than in the model group (*p* < 0.01). Notably, the medium-dose AR-ASR group showed significantly lower ICAM-1 and VCAM-1 expression than the low- and high-dose groups (*p* < 0.01), as shown in Figure 6E. These findings indicate that AR-ASR combination improves endothelial function and inhibits vascular inflammatory responses.

### 2.4. The AR-ASR Combination Inhibits Apoptosis in Aortic Cells

**Figure 7:**
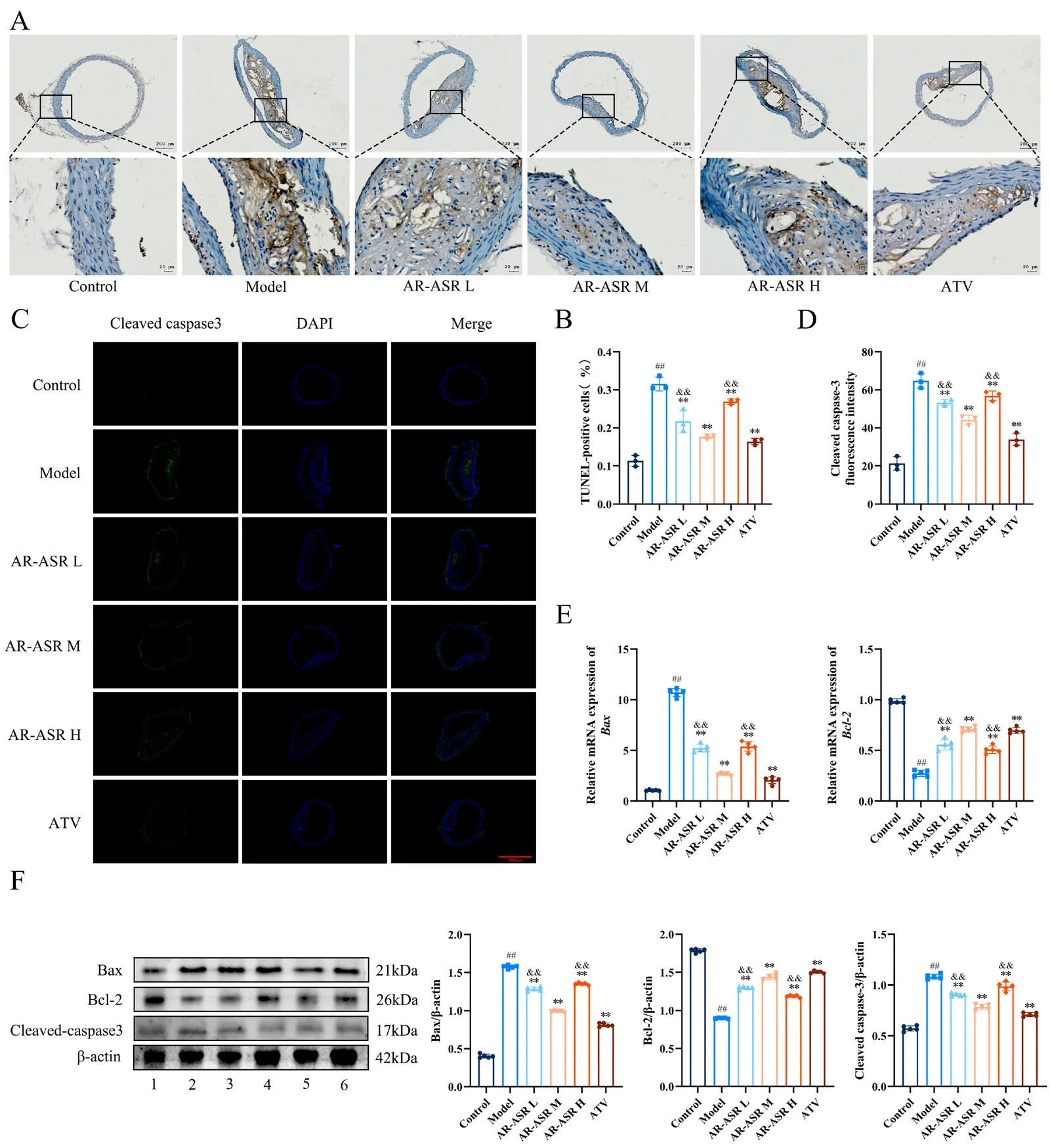
Effects of the AR-ASR Combination on Apoptosis in the Aorta ($\bar{x}$ ± *s*). (**A**) TUNEL staining of the aorta; (**B**) aortic cell apoptotic rate (TUNEL-positive cells, *n* = 3); (**C**,**D**) comparison of Cleaved caspase-3 expression in the aorta (*n* = 3); (**E**) comparison of Bax and Bcl-2 mRNA expression in the aorta (*n* = 5); (**F**) comparison of Bax, Bcl-2, and Cleaved caspase-3 protein expression in the aorta (*n* = 5). Compared to the control group: ^##^ *p* < 0.01; compared to the model group: ** *p* < 0.01; compared to the medium-dose AR-ASR group: ^&&^ *p* < 0.01.

Apoptotic death of vascular cells worsens plaque inflammation and contributes substantially to the pathogenesis and progression of AS vasculopathy [18]. We thus began by examining the influence of AR-ASR co-treatment on apoptosis within aortic cells. Figure 7A,B reveal that the model cohort exhibited a markedly higher apoptotic rate relative to the control group (*p* < 0.01). Apoptotic rates were significantly lower in the AR-ASR low-, medium-, and high-dose groups and the ATV group than in the model group (*p* < 0.01). Notably, the medium-dose AR-ASR group showed significantly lower apoptotic rates than the low- and high-dose groups (*p* < 0.05). Figure 7C,D indicate that cleaved caspase-3 levels in the aorta were significantly higher in the model group (*p* < 0.01). The ATV group and all three AR-ASR dosage groups (low, medium, and high) displayed significant reductions in cleaved caspase-3 expression relative to the model group (*p* < 0.01). Significantly reduced cleaved caspase-3 levels were observed in the intermediate AR-ASR dosage group relative to the low and high dosage groups (*p* < 0.01). These findings indicate that AR-ASR combination inhibits aortic cell apoptosis following AS.

To further elucidate the anti-apoptotic properties of AR-ASR in AS-affected aortic cells, we measured the expression of apoptosis-relevant proteins in the aorta. Figure 7E,F indicate that, compared with controls, the model group had significantly elevated Bax (mRNA and protein) and cleaved caspase-3 protein (*p* < 0.01), with concomitant significant reductions in Bcl-2 mRNA and protein (*p* < 0.01). The ATV group and all three AR-ASR dosage groups (low, medium, and high) displayed significant decreases in Bax (mRNA and protein) and cleaved caspase-3 protein, along with significant increases in Bcl-2 mRNA and protein, relative to the model group (*p* < 0.01). Significantly stronger effects were exerted by the intermediate AR-ASR dosage relative to the low and high dosage groups (*p* < 0.01).

These findings indicate that the AR-ASR combination exerts anti-apoptotic effects against AS-associated vascular cell apoptosis.

### 2.5. The AR-ASR Combination Inhibits Pyroptosis in Aortic Cells

**Figure 8:**
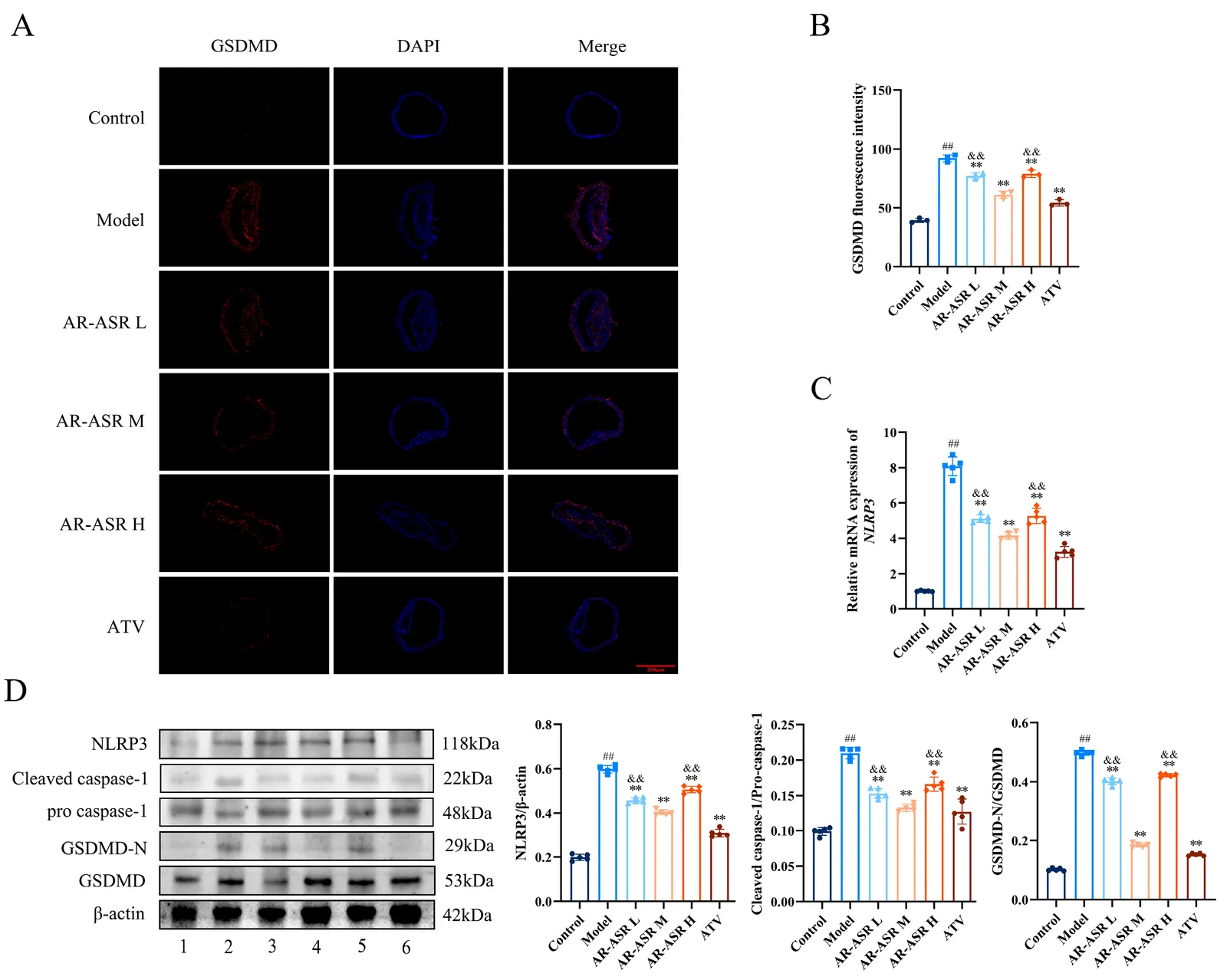
Effect of the AR-ASR Combination on Pyroptosis in the Aorta ($\bar{x}$ ± *s*). (**A**,**B**) Comparison of GSDMD expression in the aorta (*n* = 3); (**C**) comparison of NLRP3 mRNA expression in the aorta (*n* = 5); (**D**) comparison of NLRP3, Cleaved caspase-1, and GSDMD-N protein expression in the aorta (*n* = 5). Compared to the control group: ^##^ *p* < 0.01; compared to the model group: ** *p* < 0.01; compared to the medium-dose AR-ASR group: ^&&^ *p* < 0.01.

Inflammation plays an essential role in AS vasculopathy development and progression. Pyroptotic cell death, being inflammatory in nature, can aggravate plaque instability and promote rupture [19,20]. Therefore, we first investigated the effect of AR-ASR combination on pyroptosis in aortic cells following AS. GSDMD is a specific marker of pyroptosis. Upon DAMP-mediated activation, GSDMD is processed to GSDMD-N, which oligomerizes and forms membrane pores. This pore formation leads to cell lysis and the release of intracellular contents, thereby driving inflammatory activation [21]. The antibody used in this study recognizes both GSDMD and GSDMD-N, thus reflecting GSDMD activation. Immunofluorescence staining confirmed the membrane localization of GSDMD. Figure 8A,B reveal that GSDMD levels were markedly elevated in the model cohort (*p* < 0.01). Relative to the model cohort, the AR-ASR low-, medium-, and high-dose groups, along with the ATV group, exhibited markedly reduced GSDMD expression (*p* < 0.01). The medium-dose AR-ASR group also presented with significantly decreased GSDMD expression in contrast to the low- and high-dose groups (*p* < 0.05). These findings suggest that AR-ASR combination inhibits vascular cell pyroptosis in the aorta following AS.

We further examined the expression of pyroptosis-related factors. Figure 8C,D indicate that the model group had significantly higher NLRP3 (mRNA and protein), cleaved caspase-1, and GSDMD-N protein expression (*p* < 0.01). The ATV group and all three AR-ASR dosage groups (low, medium, and high) displayed significant decreases in NLRP3 (mRNA and protein), cleaved caspase-1, and GSDMD-N protein relative to the model group (*p* < 0.01). Notably, the medium-dose AR-ASR group exhibited significantly greater efficacy than the low- and high-dose groups (*p* < 0.01). These findings indicate that AR-ASR combination exerts anti-pyroptotic effects against AS-associated vascular cell pyroptosis.

### 2.6. The AR-ASR Combination Inhibits Necroptosis in Aorta Cells

**Figure 9:**
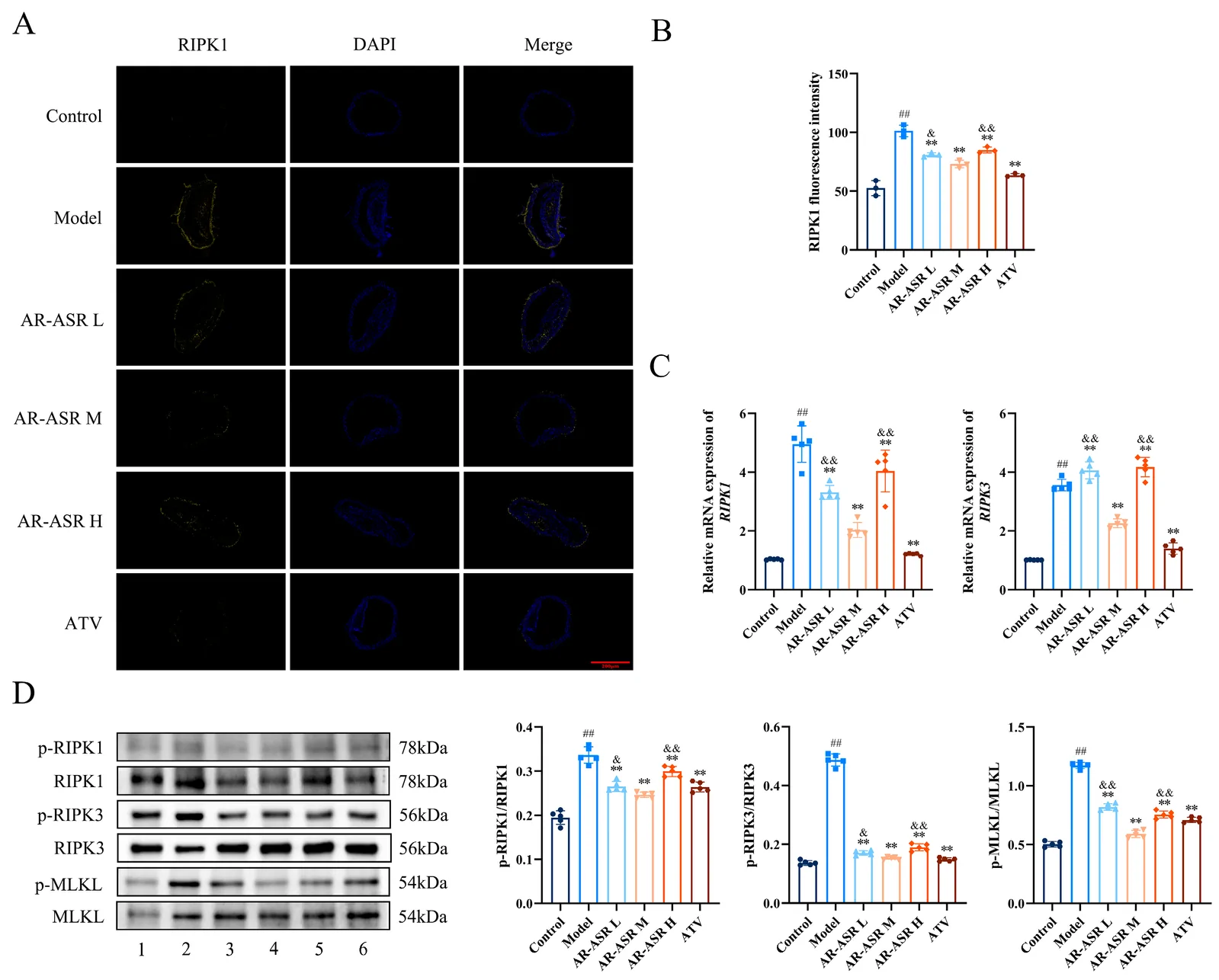
Effect of the AR-ASR Combination on Necroptosis in the Aorta ($\bar{x}$ ± *s*). (**A**,**B**) Comparison of RIPK1 expression in the aorta (*n* = 3); (**C**) comparison of RIPK1 and RIPK3 mRNA expression in the aorta (*n* = 5); (**D**) comparison of p-RIPK1, p-RIPK3, and p-MLKL protein expression in the aorta (*n* = 5). Compared to the control group: ^##^ *p* < 0.01; compared to the model group: ** *p* < 0.01; compared to the medium-dose AR-ASR group: ^&&^ *p* < 0.01, ^&^ *p* < 0.05.

Necroptosis, a second type of inflammatory cell death, is activated in AS and exacerbates both plaque instability and inflammatory responses [22]. Therefore, we further investigated the effect of AR-ASR combination on ameliorating AS vasculopathy from the perspective of necroptosis. We initially examined aortic RIPK1 expression, a marker for necroptosis. As shown in Figure 9A,B, the model cohort displayed a pronounced rise in RIPK1 levels (*p* < 0.01). The ATV group and all three AR-ASR dosage groups (low, medium, and high) displayed significant reductions in RIPK1 expression relative to the model group (*p* < 0.01). Significantly reduced RIPK1 levels were observed in the intermediate AR-ASR dosage group relative to the low and high dosage groups (*p* < 0.05). These findings indicate that AR-ASR combination inhibits vascular cell necroptosis in the aorta following AS.

We further quantified the expression of necroptosis-associated factors in the aorta. Significant upregulation of RIPK1 and RIPK3 (mRNA) as well as p-RIPK1, p-RIPK3, and p-MLKL (protein) was observed in the model group relative to controls, as depicted in panels Figure 9C,D (*p* < 0.01). The ATV group and all three AR-ASR dosage groups (low, medium, and high) displayed significant decreases in RIPK1 and RIPK3 mRNA and in p-RIPK1, p-RIPK3, and p-MLKL protein relative to the model group (*p* < 0.01). The medium-dose AR-ASR group also demonstrated significantly stronger effects in contrast to the low- and high-dose groups (*p* < 0.01). These findings indicate that AR-ASR combination exerts anti-necroptotic effects against AS-associated vascular cell necroptosis.

### 2.7. In Vivo Findings Demonstrated That the AR-ASR Combination’s Suppression of Vascular Cell PANoptosis Is Attributable to Its Inhibitory Action on the p38MAPK/p53 Pathway

The above experiments demonstrated that AR-ASR combination ameliorates AS vasculopathy and inhibits apoptosis, pyroptosis, and necroptosis in aortic cells. To further elucidate the mechanistic basis of AR-ASR’s beneficial effects on AS vasculopathy, we examined the p38MAPK/p53 pathway by immunofluorescence.

**Figure 10:**
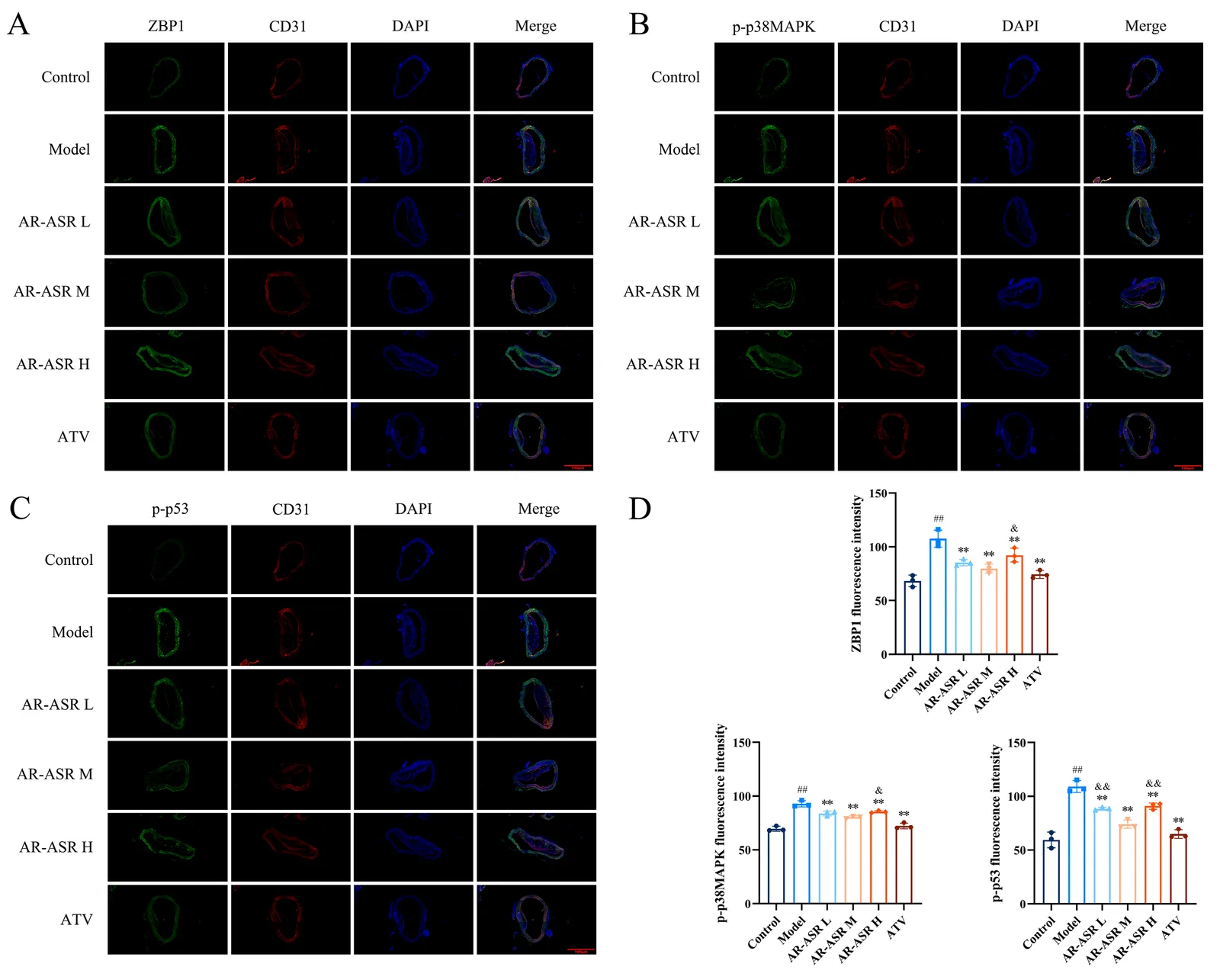
Effect of AR-ASR on aortic PANoptosis and the p38MAPK/p53 signaling pathway ($\bar{x}$ ± *s*, *n* = 3). (**A**–**D**) Comparison of the expression of the PANoptosis protein ZBP1 and p38MAPK/p53 signaling pathway proteins in aortic endothelial cells. Compared to the control group: ^##^ *p* < 0.01; compared to the model group: ** *p* < 0.01; compared to the medium-dose AR-ASR group: ^&&^ *p* < 0.01, ^&^ *p* < 0.05.

PANoptosis is driven by PANoptosome complexes; the ZBP1-PANoptosome is the most thoroughly investigated member of this group. ZBP1—a nucleic acid sensor—contains two Zα domains and exhibits selective binding to Z-DNA as well as Z-RNA [12]. ZBP1 is both an upstream sensor that can activate the PANoptosis pathway and a recognized biomarker for PANoptotic activation [11]. Studies in ZBP1-null mice reveal a marked decrease in aortic root plaque formation, supporting the notion that ZBP1 drives the development and progression of AS [23]. Therefore, we examined ZBP1 expression in aortic endothelial cells. Figure 10A,D reveal that ZBP1 levels were markedly elevated in the model cohort relative to the control group (*p* < 0.01). The ATV group and all three AR-ASR dosage groups (low, medium, and high) displayed significant reductions in ZBP1 expression relative to the model group (*p* < 0.01). Furthermore, ZBP1 levels in the medium-dose AR-ASR group were significantly lower than those in the high-dose group (*p* < 0.05). These findings indicate that AR-ASR combination inhibits PANoptosis in aortic endothelial cells following AS.

We next examined the effect of AR-ASR combination on the p38MAPK/p53 signaling pathway. Figure 10B–D reveal that the model cohort exhibited marked elevations in p-p38MAPK and p-p53 protein levels (*p* < 0.01). The ATV group and all three AR-ASR dosage groups (low, medium, and high) displayed significant reductions in p-p38MAPK and p-p53 protein expression relative to the model group (*p* < 0.01). Notably, the medium-dose AR-ASR group showed significantly lower p-p38MAPK and p-p53 expression than the low- and high-dose groups (*p* < 0.05). These findings indicate that AR-ASR combination inhibits vascular cell PANoptosis through suppression of the p38MAPK/p53 signaling pathway.

### 2.8. In Vitro Experiments Demonstrate That the AR-ASR Combination Inhibits PANoptosis in AVECs, with Effects Linked to the Inhibition of the p38MAPK/p53 Signaling Pathway Activation

The above experiments demonstrated that AR-ASR combination ameliorates AS vasculopathy and inhibits PANoptosis (apoptosis, pyroptosis, and necroptosis) in aortic cells. Since vascular endothelial injury is the initiating event and common pathological basis of atherosclerosis, to further investigate the mechanism by which AR-ASR combination ameliorates AS vasculopathy, we employed an oxidative injury model in AVECs and examined the involvement of the p38MAPK/p53 signaling pathway.

**Figure 11:**
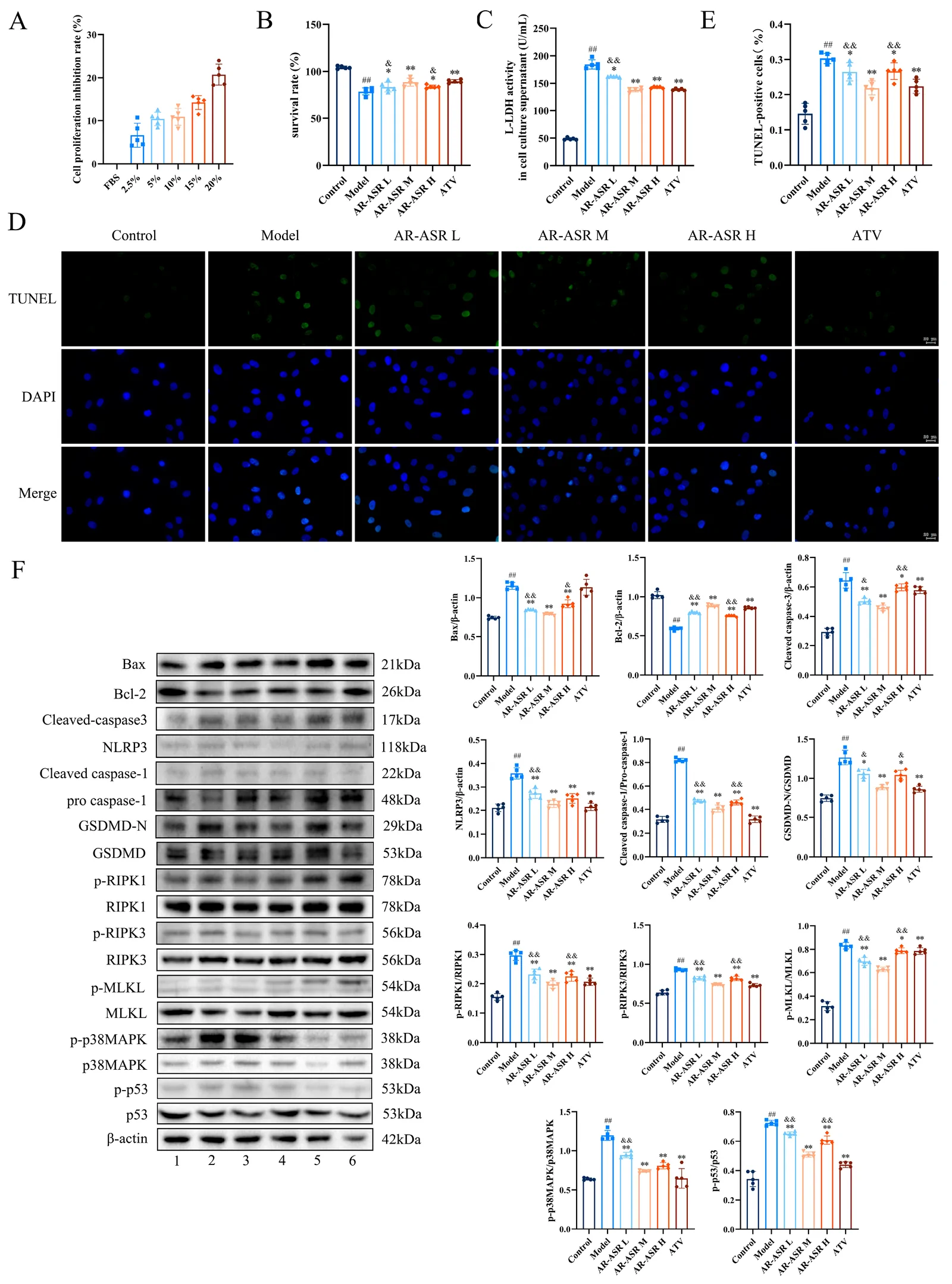
Effect of AR-ASR on PANoptosis and the p38MAPK/p53 signaling pathway in AVECs ($\bar{x}$ ± *s*, *n* = 5). (**A**) Comparison of the effects of blank serum on AVECs proliferation; (**B**) comparison of AVEC proliferation; (**C**) comparison of LDH activity in AVECs; (**D**,**E**) comparison of AVEC apoptosis; (**F**) comparison of AVEC protein expression levels of apoptosis, pyroptosis, necroptosis-related markers and p38MAPK/p53 signaling pathway components. Compared to the control group: ^##^ *p* < 0.01; compared to the model group: ** *p* < 0.01, * *p* < 0.05; compared to the medium-dose AR-ASR group: ^&&^ *p* < 0.01, ^&^ *p* < 0.05.

Using the CCK-8 assay, we initially tested the potential impact of blank serum on AVEC viability. The results showed that blank serum concentrations ranging from 2.5% to 15% did not inhibit AVEC proliferation, with cell proliferation inhibition rates of 5–15%, all below 20%. At the 20% blank serum level, cell proliferation was inhibited by 20% (Figure 11A). Therefore, subsequent experiments used drug-containing serum concentrations ranging from 2.5% to 10% to intervene with the cells.

Subsequently, we investigated the effect of the AR-ASR formulation on the proliferation of AVECs. Cell proliferation was significantly inhibited in the model group versus the blank control (*p* < 0.01). Treatment with AR-ASR drug-containing serum at low (2.5%), medium (5%), and high (10%) concentrations, as well as with atorvastatin-containing serum (10%), significantly enhanced cell proliferation relative to the model group (*p* < 0.05). The medium-concentration AR-ASR group had a higher proliferation rate than both the low- and high-concentration groups (*p* < 0.05, Figure 11B). In parallel, we quantified supernatant LDH activity as a measure of cytotoxicity. LDH release was significantly higher in the model group (*p* < 0.01). All concentrations of AR-ASR tested significantly decreased LDH levels in comparison with the model cells (*p* < 0.05). Significantly reduced LDH levels were observed with the intermediate AR-ASR treatment relative to the low-concentration group (*p* < 0.01, Figure 11C). These data demonstrate that AR-ASR alleviates oxidative stress-evoked AVEC injury.

We then explored AR-ASR’s effect on PANoptosis (apoptosis/pyroptosis/necroptosis). The model group showed significantly increased apoptotic cells (*p* < 0.01) and elevated Bax and cleaved caspase-3 proteins (*p* < 0.01), with decreased Bcl-2 expression (*p* < 0.01). Relative to the model group, treatment with low-, medium-, or high-concentration AR-ASR drug-containing serum, as well as with atorvastatin serum, significantly decreased the apoptotic cell count and lowered Bax and Cleaved caspase-3 expression (*p* < 0.05), while upregulating Bcl-2 levels (*p* < 0.01). Notably, the medium-dose AR-ASR group demonstrated significantly greater effects than the low- and high-dose groups (*p* < 0.05), as presented in Figure 11D–F. NLRP3, cleaved caspase-1, and GSDMD-N were all significantly elevated in the model group (*p* < 0.01). Administration of AR-ASR at low, medium, or high concentrations, or treatment with atorvastatin, significantly lowered the expression of these proteins relative to the model group (*p* < 0.05). NLRP3, cleaved caspase-1, and GSDMD-N levels were all lower in the medium-concentration AR-ASR group than in both the low- and high-concentration groups (*p* < 0.05; Figure 11F). Likewise, protein levels of critical necroptosis markers such as phosphorylated RIPK1, RIPK3, and MLKL were markedly elevated in the model group (*p* < 0.01). ATV and all AR-ASR concentrations tested effectively lowered the levels of these phosphorylated proteins in contrast to the model group (*p* < 0.05). A greater reduction in p-RIPK1, p-RIPK3, and p-MLKL expression was observed with the intermediate AR-ASR concentration relative to the low- and high-dose counterparts (*p* < 0.01; Figure 11F). These findings imply that the AR-ASR combination can inhibit PANoptosis in AVECs induced by oxidative stress.

Finally, we assessed the impact of the AR-ASR combination on the p38MAPK/p53 signaling axis. The model cohort exhibited markedly elevated protein levels of p-p38MAPK and p-p53 (*p* < 0.01). Treatment with low-, medium-, or high-concentration AR-ASR drug-containing serum, as well as atorvastatin serum, effectively reduced the levels of both p-p38MAPK and p-p53 relative to the model group (*p* < 0.01). The medium-concentration AR-ASR regimen induced a more pronounced decrease in p-p38MAPK and p-p53 expression than either the low- or high-concentration groups (*p* < 0.01; Figure 11F). These findings indicate that AR-ASR combination inhibits vascular endothelial PANoptosis through suppression of p38MAPK/p53 pathway activation induced by oxidative stress.

### 2.9. In Vitro Rescue Experiments Showed That AR-ASR Inhibits AVEC PANoptosis Through Suppression of p38MAPK/p53 Signaling Pathway Activation

To further explore the relationship between AR-ASR-mediated inhibition of AVEC PANoptosis and the p38MAPK/p53 signaling pathway, we investigated the effect of AR-ASR using the p38MAPK agonist Anisomycin.

**Figure 12:**
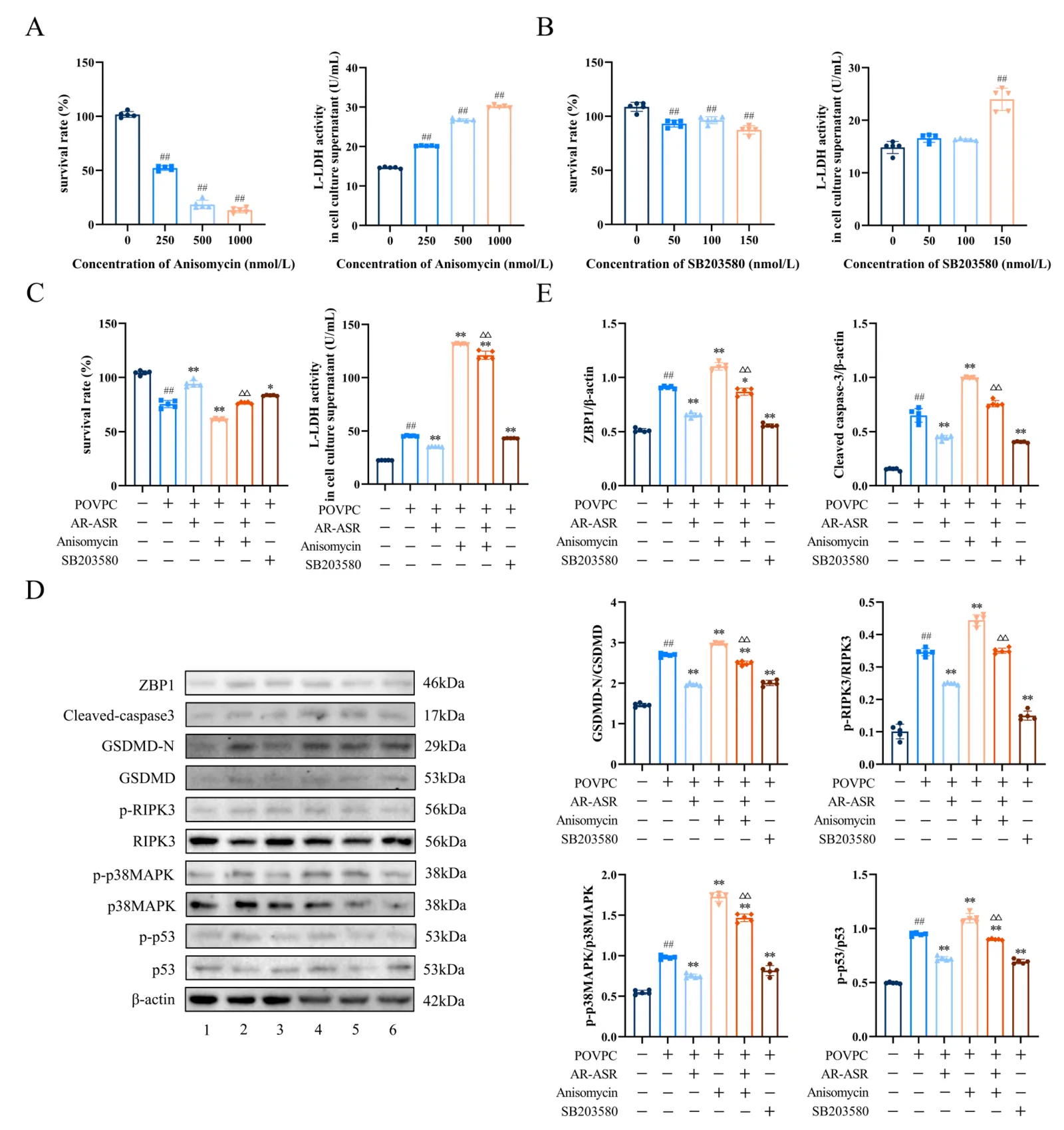
Effect of AR-ASR on AVEC PANoptosis and p38MAPK/p53 pathway in the presence of a p38MAPK agonist ($\bar{x}$ ± *s*, *n* = 5). (**A**) Evaluation of dose-dependent effects of the agonist Anisomycin on AVECs; (**B**) evaluation of dose-dependent effects of the inhibitor SB203580 on AVECs; (**C**) assessment of AR-ASR’s effect on AVEC proliferation under p38MAPK pathway agonist stimulation; (**D**,**E**) examination of AR-ASR effects on AVEC expression of PANoptosis-related and p38MAPK/p53 pathway proteins upon p38MAPK agonist treatment. 1: Control; 2: Model; 3: AR-ASR; 4: Anisomycin; 5: AR-ASR + Anisomycin; 6: SB203580. Compared to the control group: ^##^ *p* < 0.01; compared to the model group: ** *p* < 0.01, * *p* < 0.05; compared to the AR-ASR group: ^ΔΔ^ *p* < 0.01.

First, we determined the effective concentrations of the p38MAPK agonist Anisomycin and the p38MAPK inhibitor SB203580. The results showed that at a concentration of 250 nmol/L, Anisomycin significantly reduced the proliferative activity of AVECs (*p* < 0.01), with a cell damage rate of 50%; at a concentration of 100 nmol/L, SB203580 exhibited no damaging effect on AVECs (*p* < 0.01) (Figure 12A,B). Therefore, subsequent experiments were performed using 250 nmol/L Anisomycin and 100 nmol/L SB203580.

We next examined AR-ASR’s effect on AVEC proliferation with Anisomycin (p38MAPK agonist). The model group showed significantly reduced proliferation versus blank control (*p* < 0.01). AR-ASR and SB203580 significantly increased proliferation versus model (*p* < 0.05), while Anisomycin significantly decreased it (*p* < 0.01). Agonist co-treatment attenuated AR-ASR’s pro-proliferative effect (*p* < 0.01, Figure 12C). We also measured the LDH content in the cell culture medium. LDH levels in the model group were significantly elevated relative to controls (*p* < 0.01). AR-ASR and SB203580 significantly lowered LDH versus model (*p* < 0.01), whereas Anisomycin significantly raised it (*p* < 0.01). The LDH-lowering effect of AR-ASR was reduced by agonist co-treatment (*p* < 0.01, Figure 12C).

We next examined AR-ASR’s effect on PANoptosis with Anisomycin (p38MAPK agonist). The model group showed significantly increased ZBP1, Cleaved caspase-3, GSDMD-N, and p-RIPK3 (*p* < 0.01). AR-ASR and SB203580 significantly reduced these markers versus model (*p* < 0.01), whereas Anisomycin significantly increased them (*p* < 0.01). Co-treatment with the agonist weakened AR-ASR’s suppression of these PANoptosis markers (Figure 12D,E).

We finally assessed AR-ASR’s modulation of the p38MAPK/p53 axis under p38MAPK activation by Anisomycin. The model group showed markedly higher p-p38MAPK and p-p53 levels (*p* < 0.01). AR-ASR and SB203580 significantly lowered these levels versus model (*p* < 0.01), while Anisomycin significantly raised them (*p* < 0.01). AR-ASR’s suppression of p-p38MAPK and p-p53 was weakened by agonist co-treatment (*p* < 0.01, Figure 12D,E).

These results indicate that AR-ASR combination inhibits oxidative-stress-induced AVEC PANoptosis through suppression of p38MAPK/p53 signaling pathway activation.

## 3. Discussion

Apoptosis, pyroptosis, and necroptosis, as major RCD modalities, are implicated in the development and progression of AS vasculopathy [20,22]. Accumulating evidence suggests extensive crosstalk among these RCD pathways [24,25]. Taabazuing et al. showed that caspase-1 can activate caspase-3/7 and induce apoptosis in the absence of GSDMD, while caspase-3/7 can specifically inhibit pyroptosis by cleaving GSDMD at sites distinct from those cleaved by inflammatory caspases [12]. Tsuchiya et al. confirmed that caspase-1 can cleave and activate caspase-3 to induce apoptosis in the absence of GSDMD, indicating a bidirectional regulatory mechanism linking apoptosis and pyroptosis [26]. Necroptotic signaling via RIPK3-MLKL has been reported to activate the NLRP3 inflammasome and augment IL-1β release [13]. In addition, RIPK3 can activate the NLRP3 inflammasome and induce pyroptosis when MLKL is not present [27]. These studies illustrate extensive crosstalk among apoptosis, pyroptosis, and necroptosis.

PANoptosis is an emerging RCD modality characterized by the integration of apoptosis, pyroptosis, and necroptosis [11] and is importantly involved in inflammatory cell death. Its critical role in the pathophysiology of atherosclerosis has been increasingly recognized [12,28]. Caspase activation is essential for initiating apoptosis. Cleaved caspase-3 functions as a core component of the apoptotic cascade, and its regulation involves Bcl-2 and Bax, which are interconnected and reciprocally constrained throughout apoptosis [29]. Apoptosis is known to drive AS development and progression via its effects on vascular endothelial injury and inflammation [30]. NLRP3 inflammasome activation and caspase-1 are the critical molecular events underlying pyroptosis [31]. NLRP3 activation triggers cleavage of pro-caspase-1 to active caspase-1, which in turn processes GSDMD to its N-terminal membrane-pore-forming fragment. This cascade induces pyroptosis and the release of IL-1β and IL-18, thereby promoting AS pathogenesis and progression [20,31]. RIPK1 and RIPK3 bind through their RHIM domains to initiate necroptosis, inducing RIPK3 autophosphorylation, recruiting and phosphorylating MLKL, and forming the necrosome. The resultant membrane damage leads to necroptotic cell death, which plays a role in inflammatory responses [32]. Necroptotic activation in atherosclerotic plaques has been documented, with RIPK1, RIPK3, and p-MLKL markedly elevated in unstable and carotid plaques. Inhibition of this pathway has been shown to reduce AS lesion development [33,34]. The PANoptosome, assembled from apoptosis, pyroptosis, and necroptosis pathway components, serves as a common molecular platform that executes these three cell death modalities and can be activated by multiple atherosclerosis-related factors [35]. PANoptosis is mediated by PANoptosomes, and four major types have been identified: ZBP1, AIM2, RIPK1, and NLRP12 PANoptosome complexes [36]. Among these, the ZBP1-PANoptosome complex is the best studied. ZBP1 acts as an upstream signaling sensor that activates the PANoptosis pathway and regulates PANoptosis [11]. ZBP1 activation promotes NLRP3 inflammasome activation through the RIPK3/caspase-8 axis. RHIM-domain-mediated ZBP1-RIPK3 complex formation enables RIPK1 recruitment and the induction of necroptosis [37].

In this study, bioinformatics analysis suggested that AR-ASR combination may interfere with the progression of AS by regulating PANoptosis (apoptosis, pyroptosis, and necroptosis). To identify its core mechanism, we performed target intersection analysis and identified seven core Hub genes (ARCH), including TP53, TNF, and MAPK14. KEGG enrichment analysis further pointed to the MAPK and p53 signaling pathways. To further explore the role of AR-ASR in regulating the p38MAPK/p53 pathway in AS, we assessed the binding of its major active components to MAPK14 and p53 using molecular docking and MD simulations. Calycosin-7-O-β-D-glucoside, ferulic acid, senkyunolide I, ononin, calycosin, astragaloside IV, formononetin, and astragaloside I all displayed favorable binding to these targets. These findings support the notion that AR-ASR may inhibit PANoptosis and attenuate AS through these interactions. Based on these findings, this study proposes a core scientific hypothesis: AR-ASR combination specifically regulates the p38MAPK/p53 signaling axis by targeting the aforementioned core gene network, thereby modulating PANoptosis and influencing the progression of atherosclerosis. This study thus explored the action of AR-ASR on AS vasculopathy via PANoptosis, with its mechanism further investigated using in vitro cellular models.

In this study, the established AS model successfully developed aortic intimal hyperplasia and atherosclerotic plaque formation, exhibiting typical AS lesions. The model group also showed significantly higher plasma TC, TG, LDL-c, ET-1, and inflammatory cytokines (TNF-α, IL-1β, IL-18), with lower HDL-c, decreased aortic eNOS, and increased aortic ICAM-1 and VCAM-1. These findings indicate that the model successfully recapitulated AS-like lesions, accompanied by dyslipidemia, vascular dysfunction, and vascular inflammatory responses. Following AR-ASR combination intervention, aortic intimal hyperplasia was attenuated. Moreover, the treatment improved lipid profiles and vascular endothelial function, reduced plasma inflammatory cytokine levels, and inhibited aortic adhesion molecule expression. These findings suggest that AR-ASR combination inhibits aortic intimal hyperplasia and exerts regulatory effects on lipid metabolism, endothelial function, and inflammatory responses.

We further investigated the effect of AR-ASR combination on regulating PANoptosis in the aorta following AS. Immunofluorescence staining demonstrated a marked increase in apoptotic cells in aortic tissue from atherosclerotic model mice, along with increased expression of cleaved caspase-3 (an apoptosis marker), GSDMD (a pyroptosis marker), and RIPK1 (a necroptosis marker). AR-ASR treatment reduced apoptotic cell numbers and lowered cleaved caspase-3, GSDMD, and RIPK1 expression. These findings indicate that AR-ASR combination inhibits PANoptosis in the aorta following AS. Meanwhile, detection of ZBP1, an upstream signaling sensor of the PANoptosis pathway, revealed that ZBP1 expression in aortic endothelial cells was significantly increased after AS. AR-ASR combination inhibited ZBP1 expression, suggesting that AR-ASR suppresses ZBP1-mediated PANoptosis following AS. We further examined the expression of apoptosis-, pyroptosis-, and necroptosis-related factors in the aorta. AS was associated with increased cleaved caspase-3 and Bax, decreased Bcl-2, and elevated NLRP3, cleaved caspase-1, GSDMD-N, as well as p-RIPK1, p-RIPK3, and p-MLKL. AR-ASR treatment reduced cleaved caspase-3 and Bax, increased Bcl-2, and suppressed both pyroptotic (NLRP3, cleaved caspase-1, GSDMD-N) and necroptotic (p-RIPK1, p-RIPK3, p-MLKL) markers. These results indicate that AS enhances PANoptosis in the vascular wall, promoting vascular cell injury, and that AR-ASR combination inhibits vascular wall PANoptosis following AS.

Endothelial cells play a pivotal role in AS pathogenesis, and their injury marks the first step in AS lesion formation. Therefore, we employed a POVPC-induced oxidative injury model in AVECs to further investigate the effect of AR-ASR combination on PANoptosis. POVPC treatment reduced cell proliferation, increased LDH release, and induced apoptosis, with upregulated Bax and cleaved caspase-3, downregulated Bcl-2, and increased pyroptotic (NLRP3, cleaved caspase-1, GSDMD-N) and necroptotic (p-RIPK1, p-RIPK3, p-MLKL) markers. These findings indicate that oxidative stress induces AVEC injury, suppresses cell proliferation, and triggers PANoptosis. AR-ASR combination promoted cell proliferation, reduced LDH release, attenuated apoptosis, downregulated the expression of Bax, cleaved caspase-3, NLRP3, cleaved caspase-1, GSDMD-N, p-RIPK1, p-RIPK3, and p-MLKL, and upregulated Bcl-2 expression. These findings indicate that AR-ASR combination inhibits AVEC PANoptosis.

p53 has been implicated in apoptosis, pyroptosis, and necroptosis, and is considered a potential key regulator of PANoptosis [22]. p53 translocates to mitochondria, upregulates pro-apoptotic and downregulates anti-apoptotic genes, activates the mitochondrial apoptotic pathway, and induces apoptosis [38]. p53 can upregulate caspase-1 expression and activate pyroptosis [39]. p53 also functions in necroptosis. Under oxidative stress, mitochondrial p53 accumulation and direct binding to CypD—a permeability transition pore (PTP) regulator—enhance PTP opening, leading to mitochondrial swelling and necroptotic death [40]. p38MAPK phosphorylates and activates p53. The p38MAPK/p53 pathway has been shown to induce endothelial cell apoptosis and inflammation in AS models [41]. p38MAPK can be activated by phosphorylation to form p-p38MAPK, which in turn activates p53 by phosphorylation, participating in the regulation of cellular stress responses, including inflammation [42]. To further elucidate the mechanism underlying AR-ASR-mediated inhibition of vascular cell PANoptosis, we evaluated its effects on the p38MAPK/p53 pathway in in vivo AS and in vitro AVEC oxidative injury models. The results showed that p-p38MAPK and p-p53 expression in the aorta was significantly increased after AS modeling, and AR-ASR combination inhibited this increase. In the AVEC oxidative injury model, p-p38MAPK and p-p53 expression was also significantly increased, indicating activation of the p38MAPK/p53 signaling pathway and promotion of cellular PANoptosis. AR-ASR combination inhibited p-p38MAPK and p-p53 expression and simultaneously suppressed cellular PANoptosis. These findings demonstrate that AR-ASR combination inhibits vascular endothelial cell PANoptosis through suppression of the p38MAPK/p53 signaling pathway, thereby exerting protective effects against vascular cell injury.

To further verify whether AR-ASR combination exerts its anti-PANoptotic effects by inhibiting p38MAPK/p53 signaling pathway activation, we examined the effects of AR-ASR on PANoptosis and the p38MAPK/p53 signaling pathway using the p38MAPK/p53 pathway agonist Anisomycin. The results showed that Anisomycin exacerbated AVEC injury and further increased the expression of ZBP1, cleaved caspase-3, GSDMD-N, p-RIPK3, p-p38MAPK, and p-p53, indicating that the agonist further activated PANoptosis and the p38MAPK/p53 signaling pathway. AR-ASR combination alleviated AVEC injury and inhibited the expression of ZBP1, cleaved caspase-3, GSDMD-N, p-RIPK3, p-p38MAPK, and p-p53. However, in the presence of Anisomycin, these effects of AR-ASR were attenuated. These findings demonstrate that the inhibitory effects of AR-ASR on PANoptosis and the p38MAPK/p53 signaling pathway were weakened by the agonist, confirming that AR-ASR inhibits PANoptosis through suppression of the p38MAPK/p53 signaling pathway.

Notably, the present study revealed that the dose–response relationship of AR-ASR was not simply positive and linear. Across multiple parameters—including blood lipids, inflammatory cytokines, eNOS expression, and markers of cell death—the protective effects observed in the medium-dose group were generally stronger than those in the high-dose group, exhibiting a non-monotonic dose–response curve. This phenomenon is not uncommon in multi-target herbal medicine research, and the potential underlying mechanisms may include: (1) target saturation effects—the medium dose may already suffice to suppress core pathways such as p38MAPK/p53, and further dose escalation does not enhance the inhibition of these pathways; (2) accelerated metabolism at higher doses—higher doses may induce enhanced clearance or shortened half-life of the active constituents, resulting in an area under the plasma concentration–time curve (AUC) that does not increase proportionally with the administered dose; and (3) bidirectional regulatory properties of the active components in traditional Chinese medicine—many bioactive substances exert beneficial protective effects at low-to-medium doses, whereas these effects are attenuated or even lost at high doses. Overall, although the high-dose group did not exhibit abnormal hepatic or renal function or a significant decline in cell viability, the aforementioned pharmacokinetic and pharmacodynamic factors collectively contributed to a plateau or modest decline in therapeutic efficacy at the high dose. Therefore, the findings of this study suggest that, in the context of the vascular protective application of the Astragalus membranaceus–Angelica sinensis combination, the medium dose may approximate the upper boundary of the optimal therapeutic window, and that pursuit of the maximal administered dose may not be necessary in clinical practice.

**Figure 13:**
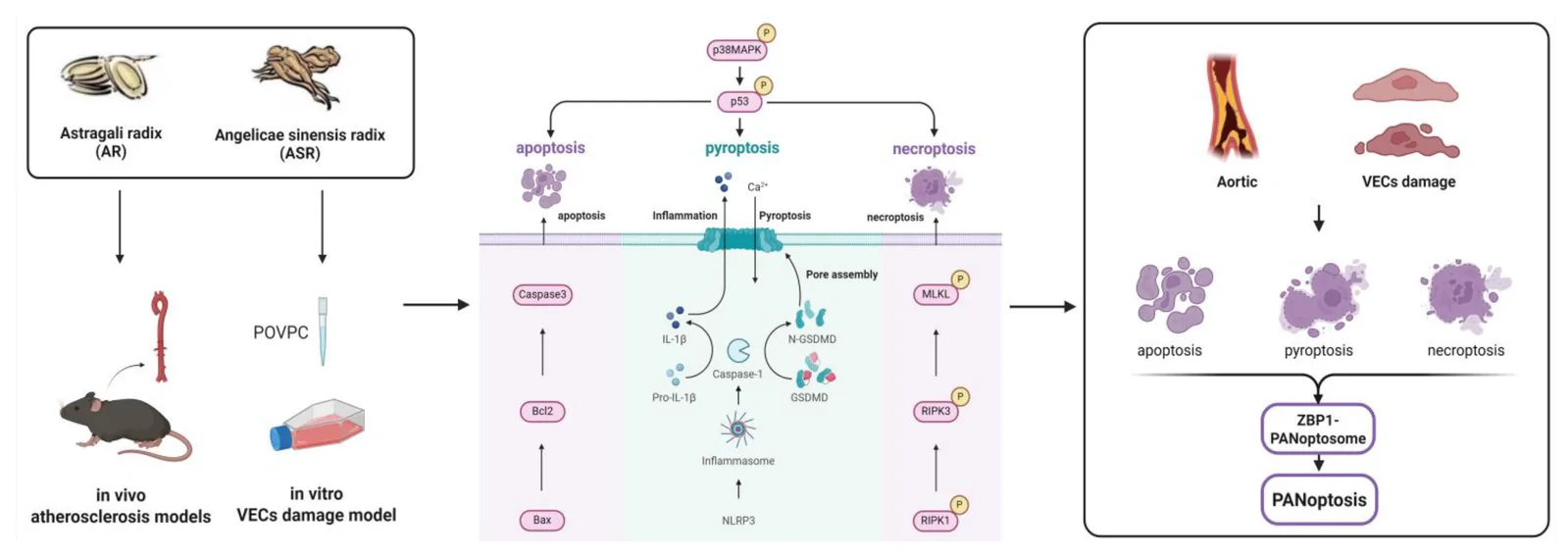
The combination of AR-ASR ameliorates AS vasculopathy through inhibition of vascular PANoptosis.

In summary, this study demonstrates that AR-ASR combination attenuates AS vasculopathy, and this effect is associated with inhibition of vascular PANoptosis through suppression of the p38MAPK/p53 signaling pathway (Figure 13). These findings suggest a new research direction for TCM intervention in AS vasculopathy via PANoptosis, and also supply a scientific basis for the application of AR-ASR combination in AS prevention and therapy.

However, the significance of vascular PANoptosis in AS vasculopathy remains to be fully elucidated. What role does vascular PANoptosis play in the evolution of AS vasculopathy? What are the mechanisms and active components through which AR-ASR combination inhibits vascular PANoptosis following AS? These questions warrant further investigation. In the future, based on the present findings, we will further explore the relationship between PANoptosis and AS vasculopathy, and utilize various vascular cell models to conduct in-depth studies on the pharmacodynamic substances and mechanisms of AR-ASR combination in ameliorating AS vasculopathy, thereby providing a scientific basis for the discovery of drugs targeting vascular pathology from traditional Chinese medicine.

## 4. Materials and Methods

### 4.1. Experimental Materials

#### 4.1.1. Animals and Animal Feed

Male ApoE^−/−^ and C57BL/6J mice, aged four to six weeks and maintained under SPF conditions, were provided by Beijing Huafukang Biotechnology Co., Ltd. (Beijing, China), with each experimental group consisting of five animals (a total of 30 mice); animal certificate number SCXK(Jing)2019-0008. Hunan Slaike Jingda Experimental Animal Co., Ltd. (Changsha, China) served as the supplier for the SPF-grade male SD rats used in this work, carrying the certificate SCXK (Xiang) 2019-0004. The animals were maintained in the SPF-grade Experimental Animal Center (Changsha, China), an institutional facility belonging to Hunan University of Chinese Medicine. The study’s animal work was conducted following ethical permission granted by the Experimental Animal Ethics Committee of Hunan University of Chinese Medicine (approval No. LL2022010401, approved on 4 January 2022). Beijing Keao Xieli Feed Co., Ltd. (Beijing, China) (certificate SCXK(Jing)2019-0003) served as the supplier for the high-fat diet, while Hunan Jiatai Experimental Animal Co., Ltd. (Yueyang, China) (certificate SCXK(Xiang)2020-0006) furnished the normal rodent chow.

#### 4.1.2. Cells

Rat aortic vascular endothelial cells (AVECs) were isolated, cultured, and passaged according to the method previously described by our group. The identity of endothelial cells was verified by immunostaining for von Willebrand factor (vWF), a marker specific to vascular endothelial cells, and cells from passages 2 to 7 were used in all experiments [43].

#### 4.1.3. Drugs

Our previous research found that combining AR and ASR in a 1:1 ratio greatly inhibited vascular intimal hyperplasia [16]. Accordingly, the 1:1 ratio of the AR-ASR combination was chosen for this investigation and assigned to three groups based on dosage: low, medium, and high. Professor Zuo Yajie identified the pharmaceutical elements for AR-ASR, which were purchased from Hunan University’s First Affiliated Hospital. Astragali Radix is the *Astragalus membranaceus* (Fisch.) Bge. var. mongholicus (Bge.) Hsiao and *Astragalus membranaceus* (Fisch.) Bge. Angelicae Sinensis Radix is the *Angelica sinensis* (Oliv.) Diels. The Drugs Museum, which falls under the administration of Hunan University of TCM’s School of Chinese Pharmacy, houses voucher specimens designated Zhou R.B. 1874 and Zhou R.B. 1862. The AR-ASR 1:1 combination, AR, and ASR were prepared using the method by our research team [16]: extracted by water heating for two rounds (the first for 2 h, and the second for 1.5 h), and the two filtrates were combined. The extracts were reduced by rotary evaporation to achieve three concentration grades—0.23, 0.46, and 0.92 g·mL^−1^ (crude drug basis)—corresponding to low, medium, and high doses, respectively, as informed by established protocols [16].

Atorvastatin not only exerts lipid-lowering effects but also possesses non-lipid-lowering actions, including anti-oxidative stress and anti-inflammatory properties [44,45]. Therefore, atorvastatin was selected as the positive control in this study. Commercial atorvastatin calcium tablets (lot H20133127, Zhejiang Lepu Pharmaceutical Co., Ltd., Taizhou, China) were suspended in isotonic saline to generate the working solution for dosing.

#### 4.1.4. Reagents

Acetonitrile (20191601) and methanol (20192217) were provided by TEDIA, USA. The Masson kit (20220420) came from Beijing Solarbio Biotechnology Co., Ltd. (Beijing, China), while ELISA kits for ET-1 (ZC-38516), TNF-α (ZC-39024), IL-1β (ZC-37974), and IL-18 (ZC-37973) were sourced from Zhuocai Biotechnology Co., Ltd., Shanghai, China. The TUNEL apoptosis detection kit (C1086) was supplied by BiYuntian Biotechnology Co., Ltd. (Shanghai, China), and the BCA Protein Assay Kit (4MFGL16GFF) was obtained from Wuhan E-Lyte Biotechnology Co., Ltd., Wuhan, China. The DAPI staining reagent (G1012) was acquired from Wuhan Saiwei Biological Technology Co., Ltd., Wuhan, China. The ECL chemiluminescence detection kit (SQ201) was acquired from Shanghai Yamei Biological Medicine Technology Co., Ltd., Shanghai, China. The RNA extraction kit (DP419) was acquired from Beijing TianGen Biotech Co., Ltd., Beijing, China. We bought the PCR amplification kit (batch number: E096) and reverse transcription kit (E047) from Shanghai JinQian Technology Co., Ltd., Shanghai, China. Wuhan PnuoSai Life Science Technology Co., Ltd. (Wuhan, China) supplied the fetal bovine serum (164210-50) and DF12 basic medium (PM150312). POVPC (870606P) was acquired from Merck, Darmstadt, Germany. We bought the CCK-8 (K1018) from APE-BIO, Houston, TX, USA. Beijing Hezi Shenggong Biotechnology Co., Ltd. (Beijing, China) supplied the LDH assay kit (AKCO003M).

eNOS antibody (27120-1-AP), Bax antibody (50599-2-Ig), Bcl-2 (26593-1-AP), p38MAPK (66234-1-Ig), MLKL (66675-1-Ig), and β-actin antibody (20536-1-AP) were obtained from Wuhan SanYing Biotechnology Co., Ltd., Wuhan, China. Cleaved caspase-3 antibody (AF7022), NLRP3 antibody (DF7438), p-MLKL (Ser358) antibody (AF7420), and p-p53 (Ser15) antibody (AF3075) were obtained from Jiangsu QingKe Biotechnology Research Center Co., Ltd., Changzhou, China. Caspase1 (pro caspase-1 + Cleaved Caspase-1) antibody (3866S), GSDMD (GSDMD + GSDMD-N) antibody (96458S), p-RIPK1 (Ser166) antibody (53286S), RIPK1 antibody (3493T), and p53 antibody (2524S) were obtained from Cell Signaling Technology (Danvers, MA, USA). p-RIPK3 (Ser232) antibody (HA721428), RIPK3 antibody (ER1901-27), and p-p38 (T180 + Y182) antibody (ER2001-52) were obtained from Hangzhou Huaan Biotechnology Co., Ltd., Hangzhou, China. ICAM-1 antibody (ab179707) and VCAM-1 antibody (ab134047) were acquired from Abcam (Cambridge, UK). ZBP1 antibody (A28338) was purchased from ABclonal Biotechnology Co., Ltd. (Wuhan, China) IgG (E-AB-1003) was acquired from Wuhan E-Lyte Biotechnology Co., Ltd. (Wuhan, China) CD31 antibody (GB113151), CY3-conjugated IgG (GB21301), Alexa Fluor 488-conjugated IgG (GB25303), IF647-Tyramide-conjugated IgG (G1232), and Alexa Fluor 594-conjugated IgG (111-585-003) were acquired from Wuhan Saiwei Biological Technology Co., Ltd., Wuhan, China.

### 4.2. Methods

#### 4.2.1. Transcriptomic and Single-Cell RNA Sequencing (scRNA-Seq) Analyses of Atherosclerosis and Bioinformatics-Based Investigation of the Anti-AS Vascular Effects of AR-ASR Compatibility

##### Identification of Differentially Expressed Genes (DEGs) in AS

For the present analysis, we obtained the atherosclerotic transcriptome dataset GSE100927 [46] from the publicly accessible GEO platform (https://www.ncbi.nlm.nih.gov/geo/ (accessed on 8 September 2026)). The DESep2 (version 1.44.0) tool in R was applied to analyze gene expression differences across 69 arterial tissues from patients affected by atherosclerotic lesions (carotid, femoral, and infrapopliteal sources) relative to 35 control arterial samples collected from subjects without atherosclerotic pathology. An adjusted P-value threshold of less than 0.05 and a |log_2_FC| greater than 1 served as the cutoffs for designating DEGs.

##### Weighted Gene Co-Expression Network Analysis (WGCNA) and Determination of Key Regulatory Genes

To explore co-expression gene sets linked to AS, we turned to the WGCNA (version 1.73) tool provided by the R software suite. Following the removal of samples identified as outliers and the exclusion of genes falling within the highest 50% of median absolute deviation values, a matrix of Pearson correlation coefficients was generated. The gene co-expression network was then created by converting the matrix into a topological overlap matrix (TOM) using a mild thresholding power (β = 5). Gene modules were subsequently delineated through hierarchical clustering using TOM dissimilarity, configured with the following criteria: a lower threshold of 100 genes per module and a deepSplit value of 3. Modules with a dissimilarity below 0.25 were merged. Lastly, core genes (Hub genes) were identified as those that had a strong correlation with the module eigengene (module membership |MM| > 0.8) [47].

##### Identification of Bioactive Constituents in AR-ASR and Forecasting Their Putative Targets in Atherosclerosis Therapy

To identify candidate bioactive molecules from AR and ASR with relevance to atherosclerosis treatment, the TCM Systems Pharmacology Database (TCMSP) was utilized. The selection process employed thresholds of oral bioavailability (OB) no less than 30% and a drug-likeness (DL) score of at least 0.18. Online resources like PubChem, SwissTargetPrediction, PharmMapper, and TargetNet were then used to estimate possible protein targets of the chosen active components. All retrieved targets were subsequently standardized to their official gene nomenclature via the UniProt (http://www.uniprot.org/ (accessed on 8 September 2026)) knowledgebase. To precisely identify core therapeutic targets, an intersection analysis was conducted between the projected AR-ASR medication targets and the DEGs and WGCNA core Hub genes obtained from earlier analyses. The results were visualized using a Venn diagram. This intersected gene set was defined as the therapeutic targets of AR-ASR in atherosclerosis (TTAA). A tripartite network depicting interactions among the drug, its bioactive constituents, and associated targets was ultimately constructed and visualized with Cytoscape (version 3.7.1) to elucidate the connections between active compounds and key molecular targets.

##### GO and KEGG Functional Enrichment Analysis

The biological relevance and potential operational pathways of the critical targets were investigated through enrichment-based approaches. We employed the clusterProfiler (version 4.12.6) toolkit within R to conduct both GO categorization and KEGG pathway evaluation. We applied a corrected *p*-value threshold of less than 0.05 to all functional enrichment assessments, thereby ensuring statistical dependability while adjusting for multiple comparisons. For clear visual display, bubble charts were utilized to illustrate the significantly enriched GO terms and KEGG pathways.

##### Association Analysis Between Core Targets and PANoptosis Pathways

We then conducted an intersection analysis of molecular targets based on the KEGG enrichment results, aiming to elucidate the precise role of TTAA in PANoptosis-related pathways. First, a core gene set for the pathways of apoptosis, pyroptosis, and necroptosis was compiled using the GeneCards (http://www.genecards.org/ (accessed on 8 September 2026)), MalaCards (https://www.malacards.org/ (accessed on 8 September 2026)) databases, and relevant literature. Subsequently, the three gene sets were individually intersected with the 105 TTAA identified in the previous analysis to identify common key targets that mediate the effects of AR-ASR in these specific modes of cell death (AR-ASR Regulated Cell Death Hub Genes, ARCH). The biological characterization and network-level relationships of the identified ARCH genes were investigated through GO/KEGG enrichment procedures, with significance set at corrected *p* < 0.05.

##### Two-Sample Mendelian Randomization Analysis Based on eQTL

We assessed potential causality from ARCH to atherosclerotic disease using two-sample Mendelian randomization (MR). This analysis respected all three basic assumptions underlying the MR design [48,49].

The exposure dataset, which included cis-eQTLs linked to the expression of ARCH genes, was obtained from the eQTLGen Consortium. This resource aggregates genotype and blood-based transcriptomic profiles from 31,684 participants of European ancestry. Obtained from the FinnGen Consortium release R8, the outcome data consist of GWAS summary statistics drawn from European cohorts, encompassing 288,638 controls and 11,189 atherosclerosis cases.

The selection of instrumental variables (SNPs) follows stringent criteria [50]. IVW-based estimation yielded the principal causal effect values in this analysis [51].

##### Single-Cell RNA Sequencing (scRNA-Seq) Data Analysis

We assessed cell-type-specific ARCH expression in the atherosclerotic plaque microenvironment by re-analyzing the GEO-derived single-cell RNA sequencing dataset GSE159677 [52]. This analysis selected sequencing data from an atherosclerotic core plaque (Atherosclerotic Core, AC; GSM4837523) and a proximal adjacent tissue (Proximal Adjacent, PA; GSM4837524) from a single patient.

After stringent quality control of the raw expression matrix—excluding cells with <200 detected genes or >5% mitochondrial reads—the dataset was normalized employing the LogNormalize algorithm. For subsequent analysis, 1500 genes with high variability were chosen. Principal component analysis (PCA) was then used to reduce linear dimensionality, and cell communities were discovered using graph clustering analysis with the appropriate number of principal components. Data visualization was conducted through t-Distributed Stochastic Neighbor Embedding (t-SNE).

To determine the biological types of the identified cell clusters, the SingleR (version 1.8.1) package, along with the HumanPrimaryCellAtlasData reference dataset, was used for automated cell type annotation. Finally, feature plots and bubble plots were employed to systematically assess the expression distributions of the seven ARCH genes (TP53, TNF, MAPK14, ATM, PYCARD, ATR, TNFRSF21) across the annotated cell types and the AC and PA tissue samples.

##### Molecular Docking

To further validate these results, we performed molecular docking of the active compounds against ARCH. Target proteins for “ARCH” and the bioactive constituents were initially screened via the UniProt database, and their respective PDB IDs were used to retrieve crystal structures from the RCSB Protein Data Bank (https://www.rcsb.org/). Water molecules and co-crystallized ligands were removed using PyMOL 2.5.0 (http://www.pymol.org/pymol (accessed on 8 September 2026)), and the structures were saved as PDB files. Receptor files were subsequently prepared by adding hydrogen atoms and converting to PDBQT format using AutoDockTools 1.5.7 (https://ccsb.scripps.edu/mgltools/downloads/ (accessed on 8 September 2026)). Ligand 2D structures (SDF format) were obtained from PubChem, subjected to energy minimization with ChemBio3D, and saved as MOL2 files, followed by conversion to PDBQT format using AutoDockTools. Molecular docking was carried out using AutoDock Vina 1.2.3 (https://vina.scripps.edu/ (accessed on 8 September 2026)). The most stable complexes were selected based on binding free energy, and docking poses were visualized with PyMOL.

##### Molecular Dynamics Simulation

Molecular dynamics (MD) simulations of the protein–ligand complexes were performed using GROMACS 2024.4. During system preparation, the protein was parameterized with the Amber99sb-ildn force field, and ligand topologies were constructed using the Sobtop (version 1.0(dev5)) tool based on the GAFF2 force field. Each complex was placed in a dodecahedral periodic box with SPC/E water molecules, and appropriate counterions were added to neutralize the system charge. The systems were then subjected to energy minimization using the steepest descent method, followed by a two-step equilibration process under NVT and NPT ensembles, each lasting 100 ps. Subsequently, a 100 ns MD simulation was conducted at a constant temperature of 300 K and constant pressure of 1 bar, with a time step of 2 fs. During the simulation, electrostatic interactions were treated using the Particle Mesh Ewald (PME) algorithm, and van der Waals interactions were handled with a force-switch function at a cutoff distance of 1.0 nm. Trajectories were saved every 50,000 steps for subsequent analyses.

Upon completion of the MD simulations, the gmx MMPBSA 1.6.4 tool was employed to quantitatively evaluate the binding free energy changes between the proteins and ligands via molecular mechanics Poisson–Boltzmann surface area (MM/PBSA) calculations. This analysis was performed on snapshots extracted from the stable phase of the MD trajectories (90–100 ns), sampled every 10 frames, using the single-trajectory approach to assess the binding free energies of the complexes, receptors, and ligands individually. The analysis not only decomposed the total binding free energy into four components—electrostatic, van der Waals, polar solvation, and nonpolar solvation contributions—but also further performed per-residue energy decomposition analysis to compute the energetic contribution of each amino acid residue within the complex to ligand binding, thereby identifying key binding sites and elucidating their impact on the overall binding free energy.

#### 4.2.2. Quality Control of the AR-ASR Combination

Calycosin-7-O-β-D glucoside, ferulic acid, Senkyunolide I, ononin, calycosin, astragaloside IV, formonnetin, and astragaloside I are the main constituents of the traditional Chinese medicinal herbs AR-ASR, and were therefore used as reference standards. To create stock solutions, precisely weighed portions of each reference standard were put into 10 mL volumetric flasks and thoroughly dissolved in methanol.

Calycosin-7-O-β-D-glucoside, ferulic acid, senkyunolide I, ononin, calycosin, astragaloside IV, formononetin, and astragaloside I are the main constituents of AR-ASR, and were therefore used as reference standards. Each reference standard was carefully weighed, dissolved in 10 mL volumetric flasks, and diluted with methanol up to the calibrated mark to yield the corresponding stock solution.

The 1:1 AR-ASR mixture, as well as the aqueous extracts of AR and ASR, were deposited in centrifuge tubes. The aqueous extract was mixed with methanol and subjected to centrifugation. Following this, the resulting supernatant was isolated and supplemented with additional methanol for subsequent analytical procedures.

The major chemical profiles of the AR-ASR (1:1) combination and its constituent AR and ASR samples were characterized and quantified by means of UPLC-MS/MS.

#### 4.2.3. Study on the Effects of AR-ASR Combination on Inhibition of Vascular Endothelial Proliferation and Pan-Vascular Lesions in ApoE^−/−^ Mice with Atherosclerosis

##### Animal Grouping and Treatment

The control arm consisted of C57BL/6J mice. ApoE^−/−^ mice subjected to atherosclerotic induction were stochastically partitioned into the model cohort, AR-ASR low-dose (AR-ASR L), AR-ASR medium-dose (AR-ASR M), AR-ASR high-dose (AR-ASR H), and atorvastatin (ATV) [53].

Table 3 shows the animal grouping and dosing details.

##### Sample Collection

Mice received an intraperitoneal injection of 2% sodium pentobarbital for anesthetic purposes. Plasma was prepared by centrifuging abdominal aortic blood, and the aorto-iliac segment—from the root to the bifurcation—was harvested as the vascular specimen.

##### Evaluation of Aortic Tissue Alterations in Mice Using Masson Staining Procedures

For Masson’s trichrome staining, the fixed aortic arch samples underwent paraffin embedding, sectioning, deparaffinization, and rehydration. Images were obtained under a microscope. Based on our group’s previously established approach [54], the intimal area, intimal thickness, intimal hyperplasia rate, and intimal thickness hyperplasia rate were measured using ImageJ (1.5.3) software.

##### Blood Lipid Profile Analysis

Plasma TC, TG, LDL-c, and HDL-c levels were assayed with an automated clinical chemistry analyzer.

##### ELISA Measurement of Plasma ET-1, TNF-α, IL-1β, and IL-18 Levels

Concentrations of ET-1, TNF-α, IL-1β, and IL-18 in plasma were determined by ELISA.

##### Western Blot Analysis of eNOS, ICAM-1, and VCAM-1 Expression in Aortic Tissue

Proteins were isolated from aortic specimens. After measuring protein content and performing denaturation, the lysates were processed through electrophoretic separation, transferred onto membranes, and incubated with blocking buffer. The blocked PVDF membranes were then exposed to primary antibodies against β-actin (1:8000), eNOS (1:500), ICAM-1 (1:1000), and VCAM-1 (1:2000). Next, incubation took place using a compatible secondary antibody labeled with horseradish peroxidase (HRP). The intensity of specific protein bands was measured based on optical density (OD) with Image Lab software (version 4.0). We calculated relative protein expression by dividing the OD value of each target protein by that of the internal control, β-actin.

##### TUNEL Staining of Apoptotic Cells in Aortic Tissue

Fixed mouse aortic arch tissues were embedded in paraffin and microtomed to 3 μm sections. Following dewaxing and rehydration, the sections were stained using the TUNEL method. ImageJ served for the enumeration of TUNEL-stained cells, and the apoptotic rate was obtained through the calculation: (TUNEL-positive cells/total cells) × 100%. The average value from three randomly selected visual fields per sample was used for statistical analysis.

##### Immunofluorescence Detection of Cleaved Caspase-3 (Apoptosis Marker), GSDMD (Pyroptosis Marker), and RIPK1 (Necroptosis Marker) in Mouse Aortic Tissue

Immunofluorescence staining was performed on fixed mouse aortic sections. After deparaffinization, hydration, and antigen retrieval, sections were blocked and incubated overnight at 4 °C with antibodies recognizing cleaved caspase-3 (1:200), GSDMD (1:200), and RIPK1 (1:1000). Following a 50 min room-temperature dark incubation with secondary antibodies conjugated to fluorophores, the sections underwent DAPI counterstaining for 10 min under dark conditions and were then coverslipped. Fluorescence imaging was performed on a standard fluorescence microscope, and the relative levels of cleaved caspase-3, GSDMD, and RIPK1 were determined by measuring their respective fluorescent signals with ImageJ (1.5.3), with results presented as signal density per unit area.

##### Double Immunofluorescence Staining of ZBP1, p-p38MAPK, and p-p53 in Mouse Aortic Endothelial Cells

Mouse aortic sections were fixed, embedded in paraffin, and sectioned as described above. The immunofluorescence staining comprised two sequential rounds. Round one: following antigen retrieval and blocking, sections were incubated overnight at 4 °C with anti-CD31 (1:2000), then with fluorophore-labeled secondary antibodies for 50 min at room temperature (dark). Round two: sections were re-incubated overnight at 4 °C with primary antibodies against ZBP1 (1:1000), p-p38 MAPK (1:1000), and p-p53 (1:1000), followed by another 50 min dark incubation with secondary fluorescent antibodies at room temperature. DAPI counterstaining was performed for 10 min in the dark, and the sections were coverslipped. Fluorescence imaging was conducted using a fluorescence microscope, and ImageJ (1.5.3) was employed to analyze the co-expression intensities of CD31 with ZBP1, p-p38 MAPK, and p-p53. The levels of the latter three markers were reported as intensity per unit area.

##### qPCR Detection of Apoptosis-Related Factors, Pyroptosis-Related Factor, and Necroptosis-Related Factors in the Aorta

RNA was isolated from aortic samples and assessed for concentration and integrity. cDNA synthesis from RNA was followed by SYBR Green qPCR amplification. Expression differences between samples were determined by the 2^−ΔΔCt^ method, relying on the threshold cycle (Ct) data from individual wells. Detailed primer information is available in Table 4.

##### Western Blot Analysis of Apoptosis, Pyroptosis, and Necroptosis Marker Protein Expression in the Aorta

The experimental protocol followed the aforementioned method. The following primary antibodies and dilution factors were applied: β-actin (1:8000), Bax (1:4000), Bcl-2 (1:2000), Cleaved caspase-3 (1:1000), NLRP3 (1:1000), GSDMD (full-length and N-terminal detection) (1:1000), p-RIPK1 (1:1000), RIPK1 (1:1000), p-RIPK3 (1:2000), RIPK3 (1:1000), p-MLKL (1:1000), and MLKL (1:1000). To obtain relative expression levels for Cleaved caspase-1, GSDMD-N, p-RIPK1, p-RIPK3, and p-MLKL, we calculated optical density ratios against pro-caspase-1, GSDMD, RIPK1, RIPK3, and MLKL, respectively.

#### 4.2.4. Study on the Anti-PANoptotic Effects of AR-ASR Compatibility in AVECs and Its Underlying Mechanisms

##### Cultivation of AVECs

AVECs were grown in DF12 media containing 10% FBS and incubated.

##### Preparation of Medicinal Serum

We randomly assigned SD rats to three groups: control, AR-ASR (1:1) combination (1.62 g/kg + 1.62 g/kg), and atorvastatin (0.9 mg/kg). After seven days of twice-daily oral gavage treatment per group, rats were anesthetized two hours post-final dose via intraperitoneal 10% sodium pentobarbital injection. Blood from the abdominal aorta was centrifuged to collect serum, namely blank control, AR-ASR (1:1)-containing, and atorvastatin-containing sera. These samples were then heat-inactivated, filter-sterilized, and stored for subsequent assays.

##### Cytotoxicity Assay of Blank Serum

Using a CCK-8 assay to evaluate any cytotoxic effects of control serum on vascular endothelial cells, we synchronized the cells at the G0 phase and then allocated them into the following experimental categories: blank group (no cells), control group (cells only), and blank serum groups (cells treated with 2.5%, 5%, 10%, 15%, or 20% blank serum). Cell proliferation inhibition percentage was computed as [(Control − Treatment)/(Control − Blank)] × 100%, with a rate under 20% indicating the non-cytotoxic concentration of blank serum.

##### Construction of AVECs Injury Model Induced by POVPC and Cell Treatment

Among the oxidized phospholipids in ox-LDL, 1-Palmitoyl-2-(5-oxovaleroyl)-sn-glycero-3-phosphocholine (POVPC) stands out as a critical mediator in the development and worsening of AS. It induces inflammatory activation and impairs endothelial functional capacity. The stability of its biological effects has rendered POVPC a standard stimulus in studies addressing endothelial injury [55]. Our prior work demonstrated that a 24 h incubation of AVECs with 35 μmol/L POVPC reliably produces a cell damage model [56]. Once cells attained 70–80% confluence, we pretreated them for three hours with either control or drug-containing serum before adding POVPC. After the pretreatment, 35 μmol/L POVPC was added for 24 h. Six AVEC groups were defined by their pretreatment conditions: Control (10% blank serum), Model (POVPC + 10% blank serum), AR-ASR L (POVPC + 2.5% AR-ASR 1:1 serum + 7.5% blank), AR-ASR M (POVPC + 5% AR-ASR 1:1 serum + 5% blank), AR-ASR H (POVPC + 10% AR-ASR 1:1 serum), and ATV (POVPC + 10% atorvastatin serum).

##### Detection of AVECs Viability by CCK-8 Method

After synchronization of the cells in the G0 phase, treatment was performed according to the groupings in “Construction of AVECs Injury Model Induced by POVPC and Cell Treatment”. Cell viability percentage was obtained via the formula: [(Treatment − Blank)/(Control − Blank)] × 100%.

##### Detection of LDH Content in Culture Medium by Colorimetric Method

Treatment was performed according to the groupings in “Construction of AVECs Injury Model Induced by POVPC and Cell Treatment”. Lactate dehydrogenase (LDH) content in the culture supernatant was quantified as specified by the kit’s manual.

##### Detection of AVECs Apoptosis by TUNEL Method

Coverslips in 24-well plates were inoculated with cells at a concentration of 2000 cells per well. Following the treatment procedures specified in “Construction of AVECs Injury Model Induced by POVPC and Cell Treatment”, the cells underwent 30 min fixation with 4% paraformaldehyde and were then washed. After a 5 min permeabilization at room temperature with buffer and subsequent washing, the cells underwent TUNEL staining and then DAPI counterstaining for 10 min in the dark. Mounted slides were examined under a fluorescence microscope for image capture. TUNEL-positive cell numbers were scored using ImageJ, and the apoptotic rate was calculated from the ratio of positive to total cells, multiplied by 100. The average value from five randomly selected visual fields per sample was used for analysis.

##### Western Blot Analysis of Apoptotic Markers, Pyroptosis Markers, Necroptotic Markers, and p38MAPK/p53 Signaling Pathway-Related Protein Expression

After treatment according to the groupings in “Construction of AVECs Injury Model Induced by POVPC and Cell Treatment”, total protein was extracted from the cells. The subsequent steps were performed in accordance with the method described in “Western Blot Analysis of Apoptosis, Pyroptosis, and Necroptosis Marker Protein Expression in the Aorta”. The expression of p-p38 was quantified as the OD ratio of p-p38 to p38, and the expression of p-p53 was quantified as the OD ratio of p-p53 to p53.

##### Effects of AR-ASR on AVEC PANoptosis and p38MAPK/p53 Signaling Pathway-Related Protein Expression in the Presence of a p38MAPK Agonist

To further analyze the effects of AR-ASR compatibility on PANoptosis and the expression of p38MAPK/p53 signaling pathway-related proteins, a p38MAPK agonist and inhibitor were used. We plated logarithmic-phase AVECs into 6-well plates. Upon attachment, cells were synchronized at the G0 stage and subsequently randomized into six groups: blank control, model control, medium AR-ASR, Anisomycin (p38MAPK agonist, 250 nmol/L), medium AR-ASR plus Anisomycin, and SB203580 (p38MAPK inhibitor, 100 nmol/L). AR-ASR medicated serum was added for 3 h of pretreatment according to the method described above, followed by treatment with POVPC, p38MAPK agonist, and inhibitor for 24 h. We employed Western blot, as previously reported, to examine the levels of PANoptosis- and p38MAPK/p53 pathway-linked proteins: ZBP1, Cleaved caspase-3, GSDMD-N, p-RIPK3, p-p38MAPK, and p-p53.

#### 4.2.5. Statistical Analysis

Data are reported as mean ± SD and were evaluated statistically using SPSS 26.0. Multi-group comparisons relied on one-way ANOVA, followed by post hoc pairwise tests—LSD for equal variances and Dunnett’s T3 for unequal variances. Statistical significance was predefined as *p* < 0.05.

## 5. Conclusions

The AR-ASR compatibility suppresses p38MAPK/p53 pathway activation, thereby inhibiting vascular endothelial PANoptosis-driven inflammatory responses and ameliorating atherosclerotic vascular pathology.

## Figures and Tables

**Table 1:** Active Compounds of AR-ASR.

Herbal	Molecule Name	OB (%)	DL
Astragali Radix	Jaranol	50.83	0.29
	isorhamnetin	49.6	0.31
	3,9-di-O-methylnissolin	53.74	0.48
	7-O-methylisomucronulatol	74.69	0.3
	9,10-dimethoxypterocarpan-3-O-β-D-glucoside	36.74	0.92
	(6aR,11aR)-9,10-dimethoxy-6a,11a-dihydro-6H-benzofurano [3,2-c]chromen-3-ol	64.26	0.42
	Bifendate	31.1	0.67
	formononetin	69.67	0.21
	isoflavanone	109.99	0.3
	Calycosin	47.75	0.24
	kaempferol	41.88	0.24
	(3R)-3-(2-hydroxy-3,4-dimethoxyphenyl)chroman-7-ol	67.67	0.26
	1,7-Dihydroxy-3,9-dimethoxy pterocarpene	39.05	0.48
	quercetin	46.43	0.28
Angelicae Sinensis Radix	beta-sitosterol	36.91	0.75
	Stigmasterol	43.83	0.76

**Table 2:** Binding Energies of AR-ASR Active Components with Core Targets.

Target	Molecule Name	Binding Energy Value (kcal·mol^−1^)
TP53	Calycosin 7-o-beta-D-glucoside	−7.3
	Ferulic acid	−5.8
	Senkyunolide I	−6.6
	Ononin	−7.5
	Calycosin	−7.0
	Astragaloside IV	−7.0
	Formononetin	−6.9
	Astragaloside I	−7.8
TNF	Calycosin 7-o-beta-D-glucoside	−8.4
	Ferulic acid	−6.3
	Senkyunolide I	−6.5
	Ononin	−8.4
	Calycosin	−8.1
	Astragaloside IV	−8.4
	Formononetin	−7.7
	Astragaloside I	−8.4
MAPK14	Calycosin 7-o-beta-D-glucoside	−8.9
	Ferulic acid	−6.2
	Senkyunolide I	−6.1
	Ononin	−8.9
	Calycosin	−7.2
	Astragaloside IV	−7.5
	Formononetin	−7.0
	Astragaloside I	−7.5
ATM	Calycosin 7-o-beta-D-glucoside	−9.4
	Ferulic acid	−6.2
	Senkyunolide I	−7.0
	Ononin	−9.4
	Calycosin	−8.2
	Astragaloside IV	−7.8
	Formononetin	−7.6
	Astragaloside I	−7.5
PYCARD	Calycosin 7-o-beta-D-glucoside	−6.7
	Ferulic acid	−4.9
	Senkyunolide I	−5.3
	Ononin	−6.5
	Calycosin	−6.2
	Astragaloside IV	−6.9
	Formononetin	−5.5
	Astragaloside I	−5.8
ATR	Calycosin 7-o-beta-D-glucoside	−9.4
	Ferulic acid	−6.2
	Senkyunolide I	−7.0
	Ononin	−9.4
	Calycosin	−8.2
	Astragaloside IV	−7.8
	Formononetin	−7.6
	Astragaloside I	−7.5
TNFRSF21	Calycosin 7-o-beta-D-glucoside	−6.9
	Ferulic acid	−5.1
	Senkyunolide I	−5.8
	Ononin	−6.7
	Calycosin	−6.3
	Astragaloside IV	−6.0
	Formononetin	−5.8
	Astragaloside I	−5.7

**Table 3:** Animal grouping and drug intervention.

Group	Pharmacological Interventions
Control	0.1 mL·10 g^−1^·d^−1^ 0.9% Sodium chloride solution by continuous gavage for four weeks
Model	0.1 mL·10 g^−1^·d^−1^ 0.9% Sodium chloride solution by continuous gavage for four weeks
AR-ASR L	1.170 g·kg^−1^·d^−1^ AR and 1.170 g·kg^−1^·d^−1^ ASR (crude drug concentration) by continuous gavage for four weeks
AR-ASR M	2.340 g·kg^−1^·d^−1^ AR and 2.340 g·kg^−1^·d^−1^ ASR (crude drug concentration) by continuous gavage for four weeks
AR-ASR H	4.680 g·kg^−1^·d^−1^ AR and 4.680 g·kg^−1^·d^−1^ ASR (crude drug concentration) by continuous gavage for four weeks
ATV	0.0012 g·kg^−1^·d^−1^ ATV by continuous gavage for four weeks

**Table 4:** Primer Sequences.

Target Gene	Primer Sequences	
*Bax*	Upstream sequence	5′-GCCTTTTTGCTACAGGGTTTCAT-3′
	Downstream sequence	5′-TATTGCTGTCCAGTTCATCTCCA-3′
*Bcl-2*	Upstream sequence	5′-TGACTTCTCTCGTCGCTACCGT-3′
	Downstream sequence	5′-CCTGAAGAGTTCCTCCACCACC-3′
*NLRP3*	Upstream sequence	5′-GCTGCGATCAACAGGCGAGAC-3′
	Downstream sequence	5′-CCATCCACTCTTCTTCAAGGCTGTC-3′
*RIPK1*	Upstream sequence	5′-CTCCGAGCAGGTCAAATTCAGA-3′
	Downstream sequence	5′-TGAGAGACCCTTCGTTTCCTTTC-3′
*RIPK3*	Upstream sequence	5′-TACTGGTGCGTCAGCGGTTC-3′
	Downstream sequence	5′-CCAGGATATCTTCTTCGAGTTCAC-3′
*GAPDH*	Upstream sequence	5′-TGAAGGTCGGTGTGAACGGATTTG-3′
	Downstream sequence	5′-TCGCTCCTGGAAGATGGTGATGG-3′

## Data Availability

The original contributions presented in this study are included in the article. Further inquiries can be directed to the corresponding authors.
